# Identification of Novel Class of Triazolo-Thiadiazoles as Potent Inhibitors of Human Heparanase and their Anticancer Activity

**DOI:** 10.1186/s12885-017-3214-8

**Published:** 2017-03-31

**Authors:** C. P. Baburajeev, Chakrabhavi Dhananjaya Mohan, Shobith Rangappa, Daniel J. Mason, Julian E. Fuchs, Andreas Bender, Uri Barash, Israel Vlodavsky, Basappa, Kanchugarakoppal S. Rangappa

**Affiliations:** 1 Laboratory of Chemical Biology, Department of Chemistry, Bangalore University, Central College Campus, Palace Road, Bangalore, 560001 India; 2 Department of Studies in Chemistry, University of Mysore, Manasagangotri, Mysore, 570006 India; 3 Department of Studies in Molecular Biology, University of Mysore, Manasagangotri, Mysore, 570006 India; 4 Adichunchanagiri Institute for Molecular Medicine, BG Nagara, Nagamangala Taluk, Mandya, district-571448 India; 5 Centre for Molecular Informatics, Department of Chemistry, University of Cambridge, Lensfield Road, Cambridge, UK; 6 Cancer and Vascular Biology Research Center, the Bruce Rappaport Faculty of Medicine, Technion, Haifa, Israel

**Keywords:** Heparanase inhibitors, triazolo-thiadiazoles, Metastasis, Anticancer activity

## Abstract

**Background:**

Expression and activity of heparanase, an endoglycosidase that cleaves heparan sulfate (HS) side chains of proteoglycans, is associated with progression and poor prognosis of many cancers which makes it an attractive drug target in cancer therapeutics.

**Methods:**

In the present work, we report the in vitro screening of a library of 150 small molecules with the scaffold bearing quinolones, oxazines, benzoxazines, isoxazoli(di)nes, pyrimidinones, quinolines, benzoxazines, and 4-thiazolidinones, thiadiazolo[3,2-a]pyrimidin-5-one, 1,2,4-triazolo-1,3,4-thiadiazoles, and azaspiranes against the enzymatic activity of human heparanase. The identified lead compounds were evaluated for their heparanase-inhibiting activity using sulfate [^35^S] labeled extracellular matrix (ECM) deposited by cultured endothelial cells. Further, anti-invasive efficacy of lead compound was evaluated against hepatocellular carcinoma (HepG2) and Lewis lung carcinoma (LLC) cells.

**Results:**

Among the 150 compounds screened, we identified 1,2,4-triazolo-1,3,4-thiadiazoles bearing compounds to possess human heparanase inhibitory activity. Further analysis revealed 2,4-Diiodo-6-(3-phenyl-[1, 2, 4]triazolo[3,4-b][1, 3, 4]thiadiazol-6yl)phenol (DTP) as the most potent inhibitor of heparanase enzymatic activity among the tested compounds. The inhibitory efficacy was demonstrated by a colorimetric assay and further validated by measuring the release of radioactive heparan sulfate degradation fragments from [^35^S] labeled extracellular matrix. Additionally, lead compound significantly suppressed migration and invasion of LLC and HepG2 cells with IC_50_ value of ~5 μM. Furthermore, molecular docking analysis revealed a favourable interaction of triazolo-thiadiazole backbone with Asn-224 and Asp-62 of the enzyme.

**Conclusions:**

Overall, we identified biologically active heparanase inhibitor which could serve as a lead structure in developing compounds that target heparanase in cancer.

**Electronic supplementary material:**

The online version of this article (doi:10.1186/s12885-017-3214-8) contains supplementary material, which is available to authorized users.

## Background

The extracellular matrix (ECM) plays a prime role in maintaining the architecture and integrity of organs and tissues [1]. Collagen, fibronectin, laminin and several growth factors and cytokines interact with heparan sulfate proteolglycans (HSPGs) in the ECM and cell surface to maintain cellular framework and function [2, 3]. Heparanase is the predominant endoglycosidase that catalyzes the cleavage of heparan sulfate (HS) polysaccharide chains in HSPGs into smaller fragments and thereby modulates the functions of HS [4–10]. Heparanase degrades the linkage between glucuronic acid and N-sulfo glucosamine residues at restricted sites of HS yielding fragments of 4-7 kDa [6]. Heparanase activity contributes to disassembly and remodeling of basement membrane and ECM resulting in upregulated cell migration and invasion and release of HS-bound growth- and angiogenesis- promoting factors [7–9]. Notably, elevated levels of heparanase are positively correlated with triggered expression of MMP-9, hepatocyte growth factor (HGF) and vascular endothelial growth factor (VEGF) that are entangled with cancer progression [11–13]. Together, these and other results critically support the intimate involvement of heparanase in tumor progression and encourage the development of heparanase inhibitors as anti-cancer therapeutics [14–16].

Several heparin/HS mimetics were demonstrated as heparanase inhibitors and some have entered clinical trials [8, 15], among these are Muparfostat (PI-88), Roneparstat (SST0001), PG545, and necuparanib (M402) [8, 15]. Muparfostat is a mixture of sulfated di- to hexasaccharides which progressed to Phase III clinical trial in post-resection hepatocellular carcinoma. It displayed significant hematologic side effects when administered with docetaxel [17, 18]. PG545, a fully sulfated hexasaccharide conjugated with a lipophilic moiety, is a dual inhibitor of heparanase and angiogenesis, currently in phase-I clinical trials in patients with advanced solid tumors ([19], https://clinicaltrials.gov/ct2/show/NCT02042781). Roneparstat, N-acetylated glycol-split heparin, is in phase I clinical trial in myeloma patients (https://clinicaltrials.gov/ct2/show/study/NCT01764880, [20]. Similarly, necuparanib (glycol-split low molecular weight heparin) is in phase-I/II trial for pancreatic cancer in combination with nab-paclitaxel and gemcitabine (https://clinicaltrials.gov/ct2/show/NCT01621243, [21]). Given the diverse effects of heparin-like compounds, these studies indicate the significance of designing chemically novel, highly selective and biologically active heparanase inhibitors to potently target various types of cancers and possibly inflammatory diseases [8, 15]. Synthesis of heparanase-inhibiting small molecules has been reported [8, 16, 22], but none was advanced to preclinical and clinical studies [8]. We have previously reported the synthesis of various heterocylces with good anticancer activity [23–29]. The current saccharide-based compounds are not specific for heparanase leaving open the question as to how much of their anti-tumor effect is due specifically to blocking heparanase activity. Herein, we screened 150 small molecules with the scaffold bearing quinolones, oxazines, benzoxazines, isoxazoli(di)nes, pyrimidinones, quinolines, benzoxazines, and 4-thiazolidinones, thiadiazolo[3,2-a]pyrimidin-5-one, 1,2,4-triazolo-1,3,4-thiadiazoles, and azaspiranes for inhibition of human heparanase enzymatic activity. Selected molecules were tested for inhibition of cell migration and invasion. The most effective compound was examined for putative binding modes against the target enzyme using molecular docking analysis.

## Methods

All solvents were of analytical grade and reagents were purchased from Sigma-Aldrich. ^1^H and ^13^C NMR spectra were recorded on a Varian and Bruker WH-200 (400 MHz) spectrometer in CDCl_3_ or DMSO-d_6_ as solvent, using TMS as an internal standard and chemical shifts are expressed as ppm. Mass spectra were determined on a Shimadzu LC-MS. High resolution mass spectra were determined on a Bruker Daltonics instrument. The elemental analyses were carried out using an Elemental Vario Cube CHNS rapid Analyzer. The progress of the reaction was monitored by TLC pre-coated silica gel G plates.

### Heparanase

Active heparanase was produced in HEK 293 cells stably transfected with the human heparanase gene construct in the mammalian pSecTag vector. The enzyme was purified and kindly provided by Dr. Yi Zhang (Eli Lilly and Company, New York, NY) [30].

### Cells

Mouse Lewis lung carcinoma (LLC; ATCC Cat. number: CRL-1642), human lung carcinoma (HCC827; ATCC Cat. number: CRL-2868), and human hepatocellular carcinoma (HepG2, Hep3B; ATCC Cat. number: HB-8065 and HB-8064, respectively) cell lines were obtained from the American Type Culture Collection and working stocks did not exceed four passages. The cell lines have recently been tested for mycoplasma contamination and authenticated using the Promega PowerPlex 16 HS kit. Cells were cultured in Dulbecco’s Modified Eagle’s Medium (DMEM) supplemented with glutamine, pyruvate, antibiotics and 10% fetal calf serum in a humidified atmosphere containing 5% CO_2_ at 37 °C.

### Real-time PCR

Total RNA was extracted with TRIzol (Sigma) and RNA (1 μg) was amplified using one step PCR amplification kit, according to the manufacturer’s (ABgene, Epsom, UK) instructions. The PCR primer sets utilized were: i) mouse heparanase - Forward: 5′ TTTGCAGCTGGCTTTATGTG 3′, Reverse: 5′ GTCTGGGCCTTTCACTCTTG 3′ (207 nucleotides); ii) mouse GAPDH - Forward: 5′ AGAACATCATCCCTGCATCC 3′, Reverse: 5′ AGCCGTATTCATTGTCATACC 3′ (348 nucleotides); iii) human heparanase - Forward: 5′ CCAGCCGAGCCACATCGCTC 3′, Reverse: 5′ ATGAGCCCCAGCCTTCTCCAT 3′ (550 nucleotides); iv) human GAPDH - Forward: 5′ ACAGTTCTAATGCTCAGTTGCTC 3′; Reverse: 5′ TTGCCTCATCACCACTTCTATT 3′ (360 nucleotides).

### Preparation of Sulphated Ceria

Hydrous cerium oxide was prepared by the hydrolysis of cerium (III) nitrate hexahydrate with 1:1 ammonia. Cerium (III) nitrate was dissolved in double distilled water. To this clear solution, dilute (1:1) aqueous ammonia was added drop-wise from a burette with vigorous stirring until the pH of the solution reached 8.

The solution was boiled for 15 min and allowed to stand overnight. The mother liquor was then decanted and the precipitate was washed several times with distilled water till it is completely free of nitrate ions which was confirmed by brown ring test. The precipitate was filtered and dried overnight at 383 K for 16 h. The hydroxide obtained was sieved to get particles of 75-100 μm mesh size and immersed in (1:1) H_2_SO_4_ solution (2 mL/g) and subjected to stirring for 4 h. Excess water was evaporated and the resulting sample was oven dried at 383 K for 16 h, calcined at 823 K for 5 h and stored in vacuum desiccator.

#### General procedure for Microwave synthesis of 4-amino-5-phenyl-4 h-1,2,4-triazole-3-thiol (2)

A mixture of methylbenzoate (1 mmol) and hydrazine hydrate (1 mmol) in 20 mL ethanol was irradiated in microwave at 700 W in a specially designed Teflon vessel containing lead acetate, until all the starting material was consumed (1-2 min, as monitored by TLC). To the above mixture (0.006 mmol) of KOH, CS_2_ (1 mmol) was added and further irradiated at 700 W for 1 min. Finally, hydrazine hydrate (2 mmol) was added drop wise to the above mixture and continued the irradiation at 700 W until a white solid appeared at the bottom (2-3 min). The lead acetate worked as a trap for H_2_S that was evolved during reaction. The solid obtained was dissolved in water (15-20 mL) and acidified with conc. HCl. The separated solid was filtered, dried and recrystallized to obtain pure *4-amino-5-phenyl-4 h-1,2,4-triazole-3-thiol.* Yield 78%, m.p. 232-234 °C; IR (KBr) _γ_/cm^−1^: 3310.07 (NH_2_ stretch), 3071.36 (aromatic CH stretch), 1472.38 (tautomeric C = S). ^1^H NMR: (400 MHz, DMSO-d6). δ:7.6-7.5 (m, 2H, ArH), 7.34-7.2 (m, 3H, ArH), 5.14 (s, 2H, NH_2_).

#### General procedure for the synthesis of 6-substituted-3-phenyl-(1,2,4)-triazolo(3,4-b)(1,3,4-thiadiazole (4a-4 h) by using SCe

To a mixture of *4-amino-5-phenyl-4 h-1,2,4-triazole-3-thiol* (1 mmol) and **(3a-h)** (1 mmol) in DMF (10 mL), SCe (20 mol%) and POCl_3_ (0.1 mmol) were added. The reaction mixture was refluxed for 10 h. Completion of the reaction was monitored by TLC and the catalyst was filtered and washed with water. Solvent was removed under reduced pressure and crushed ice was added to the concentrated mass. The pH of reaction mixture was adjusted to 8.0 using K_2_CO_3_ and KOH. The solid obtained was separated by filtration, washed with excess water, dried and recrystallized using appropriate solvent.

#### General procedure for the synthesis of 2-hydroxy-3,5-diiodo-N-(3-phenyl-5-thioxo-1H-1,2,4-triazol-4(5H)-yl)benzamide (5a) and 2-hydroxy-5-iodo-N-(3-phenyl-5-thioxo-1H-1,2,4-triazol-4(5H)-yl)benzamide (5b)

To **3a** (1 eq) in DMF, EDC (1.1 eq) and HOBt (1.1 eq) was added and stirred at room temperature for 30 min. It was followed by the addition of amine **(2)** and stirred for 2 h. After completion of the reaction, it was diluted with water and the obtained solid was filtered and re-crystallized in appropriate solvent.

#### 2,4-Diiodo-6-(3-phenyl-[1, 2, 4]triazolo[3,4-b][1, 3, 4]thiadiazol-6yl)phenol (4a, DTP)

Yellow colored solid; ^1^H NMR (400 MHz, DMSO-d_6_) 8.37-8.35 (d, 2H), 8.26 (s, 1H), 7.85 (s, 1H), 7.69-7.63 (m, 2H), 7.54-7.52 (d, 1H), 4.92 (s, 1H); ^13^C NMR (DMSO-d_6_); 165.53, 154.53, 149.29, 148.83, 140.98, 137.51, 133.83, 132.45, 129.11, 128.64, 123.10, 122.44, 120.72, 96.18, 85.11; HRMS Calcd 568.840; Found: 568.840 (M + Na)^+^; Anal. Calcd for C_15_H_8_I_2_N_4_OS: C, 32.99; H, 1.48; N, 10.26; Found: C, 33.00; H, 1.49; N, 10.28.

#### 6-(4-(1H-Imidazol-1-yl)phenyl)-3-phenyl-[1, 2, 4]triazolo[3,4-b][1, 3, 4]thiadiazole (4b)

Pale yellow colored solid; ^1^H NMR (400 MHz, DMSO-d6) *δ*: 8.46-8.44 (d, 2H), 7.81-7.77 (m, 2H), 7.53-7.49 (m, 3H), 7.39-7.34 (m, 3H), 7.27-7.24 (m, 2H); ^13^C NMR (DMSO-d_6_); 161.55, 149.29, 148.53, 140.98, 137.18, 137.11, 133.83, 132.48, 131.97, 129.11, 128.64, 128.18, 123.10, 122.43, 120.27; LCMS (MM:ES + APCI) 345.2 (M + H)^+^. Anal. Calcd for C_18_H_12_N_6_S: C, 62.77; H, 3.51; N, 24.40; Found: C, 62.79; H, 3.53; N, 24.43.

#### 4-Iodo-2-(3-phenyl-[1, 2, 4]triazolo[3,4-b][1, 3, 4]thiadiazol-6-yl)phenol (4c, ITP)

Yellow colored solid; ^1^H NMR (400 MHz, DMSO-d6) *δ*: 8.44-8.42 (d, 2H), 8.08-8.06 (d, 2H), 8.02-8.00 (m, 1H), 7.95-7.91 (m, 1H), 7.71 (s, 1H), 7.16-7.14 (d, 1H), 4.92 (s, 1H); ^13^C NMR (DMSO-d6) *δ*: 164.19, 159.73, 152.02, 147.46, 138.26, 133.27, 131.64, 129.40, 127.70, 124.93, 120.48, 119.82, 118.66, 88.23; HRMS Calcd 442.943; Found: 442.943 (M + Na)^+^; Anal. Calcd for C_15_H_9_IN_4_OS: C, 42.87; H, 2.16; N, 13.33; Found: C, 42.89; H, 2.17; N, 13.35.

#### 6-(((R)-Tetrahydro-2H-pyran-2-yl)(phenyl)methyl)-3-phenyl-[1, 2, 4]triazolo[3,4-b][1, 3, 4]thiadiazole (4d)

White colored solid; ^1^H NMR (400 MHz, DMSO-d6) *δ*: 8.25-8.16 (d, 2H), 8.06 (m, 1H), 7.78-7.76 (m, 1H), 7.62-7.60 (m, 1H), 7.27-7.15 (m, 4H), 4.58-4.53 (m, 2H), 3.88-3.84 (m, 2H), 1.78-1.73 (m, 4H), 1.50-1.45 (m, 2H); ^13^C NMR (DMSO-d6) *δ*: 164.56, 149.30, 143.93, 141.04, 137.49, 132.82, 132.41, 130.23, 129.10, 128.10, 120.70, 80.11, 71.09, 43.59, 30.41, 30.33, 21.48; LCMS (MM:ES + APCI) 377.2 (M + H)^+^; Anal. Calcd for C_21_H_20_N_4_OS: C, 67.00; H, 5.35; N, 14.88; Found: C, 67.02; H, 5.37; N, 14.90.

#### 2-(3-Phenyl-[1, 2, 4]triazolo[3,4-b][1, 3, 4]thiadiazol-6yl)-1-p-tolylethanone (4e)

White colored solid; ^1^H NMR (400 MHz, DMSO-d6) *δ*: 8.43-8.41 (m, 2H), 8.03-7.99 (m, 3H), 7.92 (m, 1H), 7.69 (m, 1H), 7.40-7.38 (m, 2H), 4.1 (s, 2H), 2.42 (m, 3H); ^13^C NMR (DMSO-d6) *δ*:192.83, 164.18, 159.42, 151.99, 146.87, 137.47, 132.28, 130.26, 125.66, 123.38, 121.01, 120.89, 48.13, 21.13; HRMS Calcd 357.078; Found: 357.078 (M + Na)^+^. Anal. Calcd for C_18_H_14_N_4_OS: C, 64.65; H, 4.22; N, 16.75; Found: C, 64.67; H, 4.25; N, 16.77.

#### 6-(3-4-Dimethoxybenzyl)-3-phenyl-[1, 2, 4]triazolo[3,4-b][1, 3, 4]thiadiazole (4f)

Yellow colored solid; ^1^H NMR (400 MHz, DMSO-d6) *δ*: 8.2 (d, 2H), 7.6-7.4 (m, 3H), 7.0 (s, 1H), 6.9 (d, 2H), 4.4 (s, 2H), 3.8 (s, 6H); LCMS (MM:ES + APCI) 353.2 (M + H)^+^; Anal.Calcd for C_18_H_16_N_4_O_2_S: C, 61.35; H, 4.58; N, 15.90; Found: C, 61.39; H 4.59; N, 15.93.

#### 3-(3-Phenyl--[1, 2, 4]triazolo[3,4-b][1, 3, 4]thiadiazol-6-yl-)phenol (4 g)

White colored solid; ^1^H NMR (400 MHz, DMSO-d6) *δ*: 8.32-8.31 (m, 2H), 8.13 (s, 1H), 7.94-7.87 (m, 3H), 7.65-7.59 (m, 2H), 7.46 (m, 1H), 4.91 (s, 1H); LCMS (MM:ES + APCI) 295.2 (M + H)^+^; Anal. Calcd for C_15_H_10_N_4_OS: C, 61.21; H, 3.42; N, 19.04; Found: C, 61.23; H, 3.44; N, 19.07.

#### 3-Phenyl-6-styryl-[1, 2, 4]triazolo[3,4-b][1, 3, 4]thiadiazole (4 h)

White colored solid; ^1^H NMR (400 MHz, DMSO-d6) *δ*: 8.25-8.22(d, 2H), 7.92-7.87 (m, 2H), 7.73-7.55 (m, 4H), 7.32-7.26 (m, 2H), 6.45-6.42 (m, 2H); ^13^C NMR (DMSO-d6) *δ*: 164.84, 159.58, 153.39, 145.42, 139.89, 131.08, 131.04, 130.53, 130.42, 130.31, 129.09, 125.84, 125.47, 118.08, 116.18, 115.90; HRMS Calcd 327.067; Found: 327.067 (M + Na)^+^; Anal. Calcd for C_17_H_12_N_4_S: C, 67.O8; H, 3.97; N, 18.41; Found: C, 67.09; H, 3.99; N, 18.44.

#### 2-Hydroxy-3,5-diiodo-N-(3-phenyl-5-thioxo-1H-1,2,4-triazol-4(5H)-yl)benzamide (5a, HTP)

Pale yellow colored solid; ^1^H NMR (400 MHz, DMSO-d6) *δ*: 14.64 (s, NH), 12.30 (s, NH), 8.48 (s, 1H), 8.40 (s, 1H), 8.24-8.15 (m, 3H), 7.81-7.78 (m, 2H), 4.73 (s, 1H); ^13^C NMR (DMSO-d6) *δ*:181.47, 173.23, 153.47, 147.94, 145.12, 136.38, 134.36, 131.13, 129.09, 128.78, 128.21, 126.02, 125.58, 90.79, 72.33; HRMS Calcd 586.851; Found: 586.851 (M + Na)^+^; Anal.Calcd for C_15_H_10_I_2_N_4_O_2_S: C, 31.94; H, 1.79; N, 9.93; Found: C, 31.96; H, 1.81; N, 9.93.

#### 2-Hydroxy-5-iodo-N-(3-phenyl-5-thioxo-1H-1,2,4-triazol-4(5H)-yl)benzamide (5b)

Pale yellow colored solid; ^1^H NMR (400 MHz, DMSO-d6) *δ*: 12.5 (s, NH), 8.5 (s, 1H), 8.4 (m, 1H), 8.1 (m,1H), 7.8 (m, 3H), 7.6 (m,1H), 4.6 (s, 1H); LCMS (MM:ES + APCI) 438.4 (M + H)^+^; Anal. Calcd for C_15_H_11_IN_4_O_2_S: C, 41.11; H, 2.53; N, 12.78; Found: C, 41.12; H, 2.56; N, 12.80.

Spectral data of the compounds are presented in Additional file 1: Figure S1.

#### Colorimetric heparanase assay

The assay, carried out in 96 well microplates, measures the appearance of the disaccharide product of heparanase-catalyzed fondaparinux cleavage, colorimetrically using the tetrazolium salt WST-1 [31]. Briefly, assay solutions (100 μL) are composed of 40 mM sodium acetate buffer (pH 5.0) and 100 mM fondaparinux (Arixtra) with or without increasing concentrations of inhibitor. Recombinant heparanase was added to a final concentration of 140 pM, to start the assay. The plates are incubated at 37 °C for 18 h and the reaction is stopped by the addition of 100 μL solution containing 1.69 mM 4-[3-(4-iodophenyl)-2-(4-nitrophenyl)-2H-5-tetrazolio]-1,3-benzene disulfonate (WST-1) in 0.1 M NaOH. The plates are developed at 60 °C for 60 min, and the absorbance is measured at 584 nm. In each plate, a standard curve constructed with D-galactose as the reducing sugar standard is prepared in the same buffer and volume over the range of 2–100 μM [31].

#### ECM degradation heparanase assay

The semi-quantitative heparanase assay was performed as described previously [32, 33]. Briefly, metabolically sulfate [^35^S] labeled ECM deposited by cultured endothelial cells and coating the surface of 35 mm tissue culture dishes [33], is incubated (3 h, 37 °C, pH 6.0, 1 mL final volume) with recombinant human heparanase (200 ng/mL) in the absence and presence of candidate small molecules. The ECM was also incubated (24 h, 37 °C, pH 6.0) with cell lysates (200 μg protein/dish) prepared by 3 cycles of freeze and thaw in reaction buffer, as described [32]. To evaluate the occurrence of proteoglycan degradation, the incubation medium is collected and applied for gel filtration on Sepharose 6B columns (0.9 × 30 cm). Fractions (0.2 mL) are eluted with PBS and counted for radioactivity. The excluded volume (Vo) is marked by blue dextran and the total included volume (Vt) by phenol red. Degradation fragments of HS side chains are eluted from Sepharose 6B at 0.5 < Kav < 0.8 (fractions 12-25) [32].

#### In vitro cytotoxicity assay

The antiproliferative effect of the compounds against LLC (Lewis lung carcinoma) and HepG2 (hepatocellular carcinoma) cells was determined by the MTT dye uptake method as described previously [34–36]. Briefly, cells (2.5 × 10^4^/mL) were incubated in triplicate in a 96-well plate, in the presence of varying concentrations of test compounds at a volume of 0.2 mL, for different time intervals at 37 °C. Thereafter, 20 μL MTT solution (5 mg/mL in PBS) was added to each well. After 2 h incubation at 37 °C, 0.1 mL lysis buffer (20% SDS, 50% dimethylformamide) was added and incubated for 1 h at 37 °C, and the optical density (OD) at 570 nm was measured using a plate reader.

### In vitro trans-well invasion/migration assay

Invasion of cells (LLC, HepG2) across a Matrigel™ coated membrane or migration through control uncoated inserts was assessed using 24-well plates (BD Biosciences, 8 μm pore size, insert size: 6.4 mm) according to the manufacturer’s protocol and as described earlier [37–39]. Briefly, single cell suspensions (1 × 10^6^ cells/mL) were prepared by detaching and resuspending the cells in DMEM containing 0.1% BSA. Before adding the cells, the chambers were rehydrated for 2 h in an incubator at 37 °C. The lower chambers were filled with 600 μL DMEM containing chemo-attractant (10% FBS). After seeding the cells (2 × 10^5^ in 200 μL of serum-free medium) into the upper chamber of triplicate wells with or without increasing concentrations of compounds, the chambers were incubated for 24 h (LLC) and 48 h (HepG2) at 37 °C. The non-invaded cells were removed from the upper surface of the membrane by scrubbing and cells that migrated through the filter were fixed, stained with Diff Quick solution, counted by examination of at least five microscopic fields and photographed.

## Results

### Chemical synthesis and characterization

In recent years, solid acid catalysts have gained considerable attention due to their high efficiency, eco-friendly, longer catalyst life, negligible equipment corrosion and their reusability. In present work we report the synthesis of novel 1,2,4-triazolo-1,3,4-thiadiazoles bearing compounds via sulfated ceria mediated cycalization reaction [40–42]. Initially we synthesized the sulphated ceria (SCe) catalyst as reported previously [43]. The powdered X-ray diffraction (PXRD), Burner- Ememett-Teller (BET) and Scanning Electron microscope patterns of SCe matched with the standard material.

**Scheme 1:**
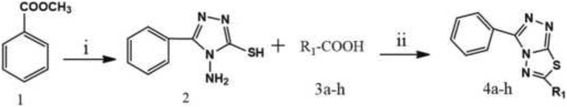
Schematic representation of new heparanase inhibitors used in this study. **i)** hydrazine hydrate, ethanol, MWI; CS_2_ and KOH, 5-6 min at 700 watt; **ii)** SCe (20 mol%), DMF, 10 h

The experimental strategy for the synthesis of starting material 4-amino-5-phenyl-4 h-1,2,4-triazole-3-thiol **(2)** was achieved by Microwave method as reported recently (Scheme 1 **, i**) [36]. In order to synthesize the novel 1,2,4-triazolo-1,3,4-thiadiazoles, we focused on the efficiency of SCe in cyclisation reaction. To optimise the reaction conditions, we attempted reaction in the combination of **2** and 3-oxo-3-(p-tolyl)propanoic acid **(3e)** as a model reaction in different concentrations of SCe and the results are summarised in Additional file 1: Table S1. The ideal system for the cyclization was found to be 20 mol% of SCe in DMF (Additional file 1: Table S1, entry 8). We also observed incomplete conversions, when SCe was lower than 20 mol%, despite of longer reaction time. From the above reaction, we examined the generality of method by synthesizing series of 1,2,4-triazolo-1,3,4-thiadiazoles molecules (Scheme 1, ii).

### Influence of SCe on cyclization

The modification of SCe with anions such as sulphate ions forms a super acidic catalyst which effectively catalyses the cyclization. Majority of reactions completed within 10 h and undissolved SCe was separated by simple filtration and finally furnished the product in good yield (Additional file 1: Table S1).

### Plausible mechanism

**Scheme 2:**
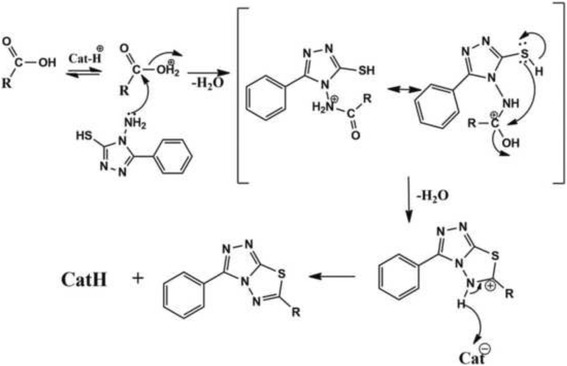
Plausible mechanism of cyclization and synthesis of title compounds

The First step involves the protonation of acid followed by dehydration and simultaneous attack of nitrogen lone pair to the electron deficient acylium ion to form an intermediate. In the second step, the intermediate undergoes neighboring group participation with nucleophilic sulphur, which leads to the formation of C-S bond by the elimination of water molecule (Scheme 2). Finally, deprotonation results in the formation of the title products **(4a-h).**

### Re-usability of acid catalyst system

Experiment was performed to study the recyclability of the SCe system employing **2** with **3e** to yield compound **4e** (Scheme 1). After each run, catalyst was removed by filtration from the reaction mixture, washed thoroughly with acetone, dried and activated at 823 K and taken for next cycle. We observed significant reduction in the yield of the product after second run (Additional file 1: Table S2). It is important to note that this system is recyclable twice with the isolated yields above 70%.

**Scheme 3:**
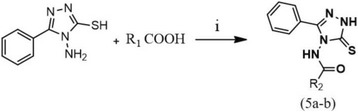
Synthetic scheme for the preparation of N-amino-triazole-amides. i) HOBt/EDC, DMF, RT, 2 h. R_1_ = **3a, 3c**

Further, we synthesized the amide derivatives of **2** with corresponding mono and di iodo salicylic acid **(3a** and **3c)** via HOBt/EDC amide formation reactions (Scheme 3) which resulted in the products 2-hydroxy-3,5-diiodo-N-(3-phenyl-5-thioxo-1H-1,2,4-triazol-4(5H)-yl)benzamide **(5a)** and 2-hydroxy-5-iodo-N-(3-phenyl-5-thioxo-1H-1,2,4-triazol-4(5H)-yl)benzamide **(5b)**. The compounds obtained were characterized by ^1^H NMR, ^13^C NMR, and mass spectral analysis (Additional file 1: Figure S1 – Spectral data). Detailed chemical characterization of the newly synthesized compounds is provided in the ‘methods’ section.

### In vitro screening of the small molecule library for inhibition of the catalytic activity of human heparanase

**Fig. 1:**
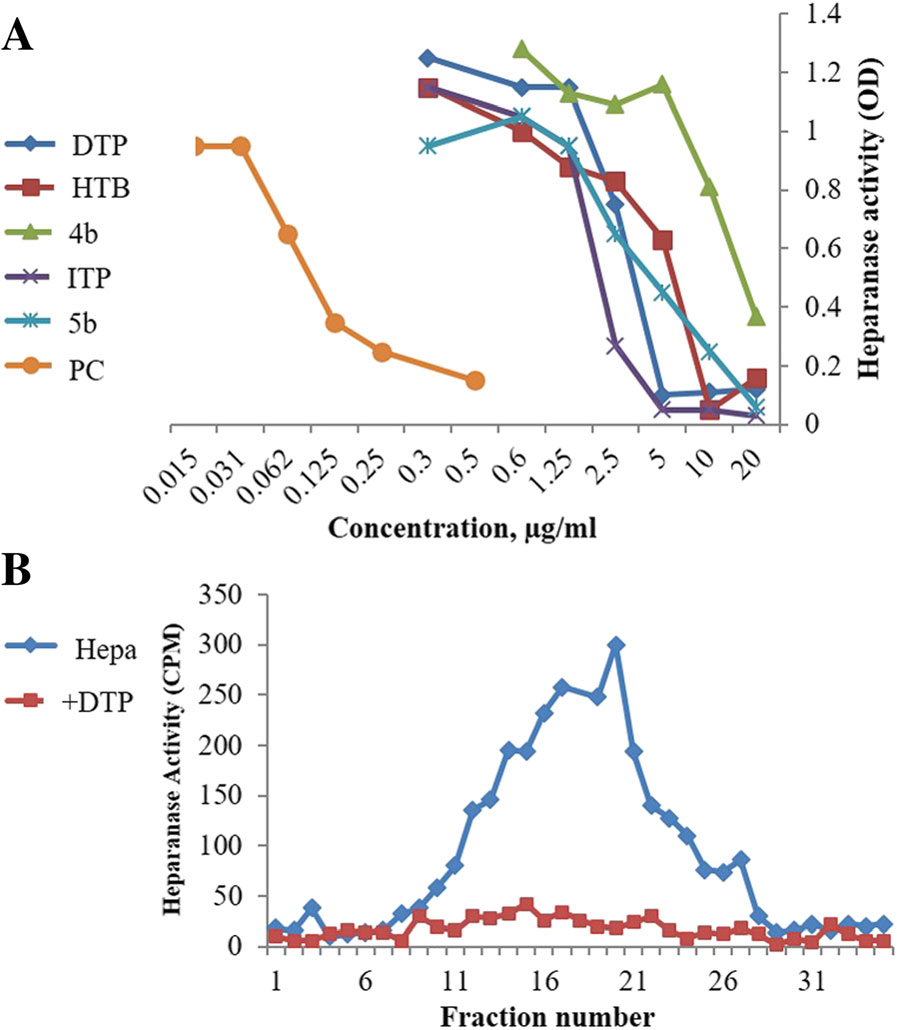
**a** Screening of compounds for inhibition of heparanase enzymatic activity applying the Fondaparinux heparanase assay. PC, positive control = N-(4-{[4-(1H-Benzoimidazol-2-yl)-arylamino]-methyl}-phenyl)-benzamide [22]. **b** Lead molecules which exhibited inhibitory activity against human heparanase were validated using a semi-quantitative assay that measures release of radioactive heparan sulfate fragments from an insoluble extracellular matrix as described in ‘Methods’ section. Briefly, sulfate [^35^S] labeled ECM was incubated (6 h, 37 °C, pH 6.0) with recombinant human heparanase (200 ng/mL) in the absence and presence of 10 μg/mL of the test compounds. Sulfate labeled material released into the incubation medium was subjected to gel filtration on Sepharose 6B. Compound DTP effectively inhibited the cleavage and release of heparan sulfate degradation fragments

Initially we screened the entire library of small molecules with diverse structures for their in vitro inhibitory activity against recombinant human heparanase at different concentrations up to 20 μg/mL. We used a 96-well based colorimetric assay that measures the ability of recombinant heparanase to degrade fondaparinux (heparin derived pentasaccharide) in solution [31]. The assay measures the appearance of a disaccharide product of fondaparinux cleavage, using the tetrazolium salt WST-1 [31]. Compounds bearing triazolo-thiadiazole backbone displayed significant inhibitory activity, 2,4-Diiodo-6-(3-phenyl-[1, 2, 4]triazolo[3,4-b][1, 3, 4]thiadiazol-6yl)phenol (DTP) being the lead and consistently active structure followed by 2-hydroxy-3,5-diiodo-N-(3-phenyl-5-thioxo-1H-1,2,4-triazol-4(5H)-yl)benzamide (HTP) and 4-iodo-2-(3-phenyl-[1, 2, 4]triazolo[3,4-b][1, 3, 4]thiadiazol-6-yl)phenol (ITP) (Fig. 1a).

In order to better resemble the in vivo situation, we applied as substrate metabolically sulfate [Na_2_^35^SO_4_] labeled extracellular matrix (ECM) deposited by cultured endothelial cells [32]. This naturally produced substrate closely resembles the subendothelial basement membrane in its composition, biological function and barrier properties. Years of experience revealed that compounds that effectively inhibit the enzyme in this assay are also effective in preclinical animal models [20, 44, 45]. This semi-quantitative assay measures release of radioactive heparan sulfate (HS) degradation fragments from an insoluble extracellular matrix (ECM) that is firmly bound to a culture dish [32, 33]. Briefly, the ECM substrate is incubated with recombinant human heparanase in the absence and presence of candidate small molecules. The incubation medium is collected and subjected to gel filtration on Sepharose 6B. Degradation fragments of heparan sulfate side chains are eluted at 0.5 < Kav < 0.8, whereas nearly intact HSPG is eluted next to the void volume [32]. As demonstrated in Fig. 1b, compound DTP (10 μg/mL) completely inhibited the release of heparan sulfate degradation fragments. The other structural analogs were less effective (not shown). Thus, we have identified heparanase-inhibiting lead compound from a random screen of bioactive compounds.

### Heparanase activity in various hepatocellular and lung carcinoma cell lines

**Fig. 2:**
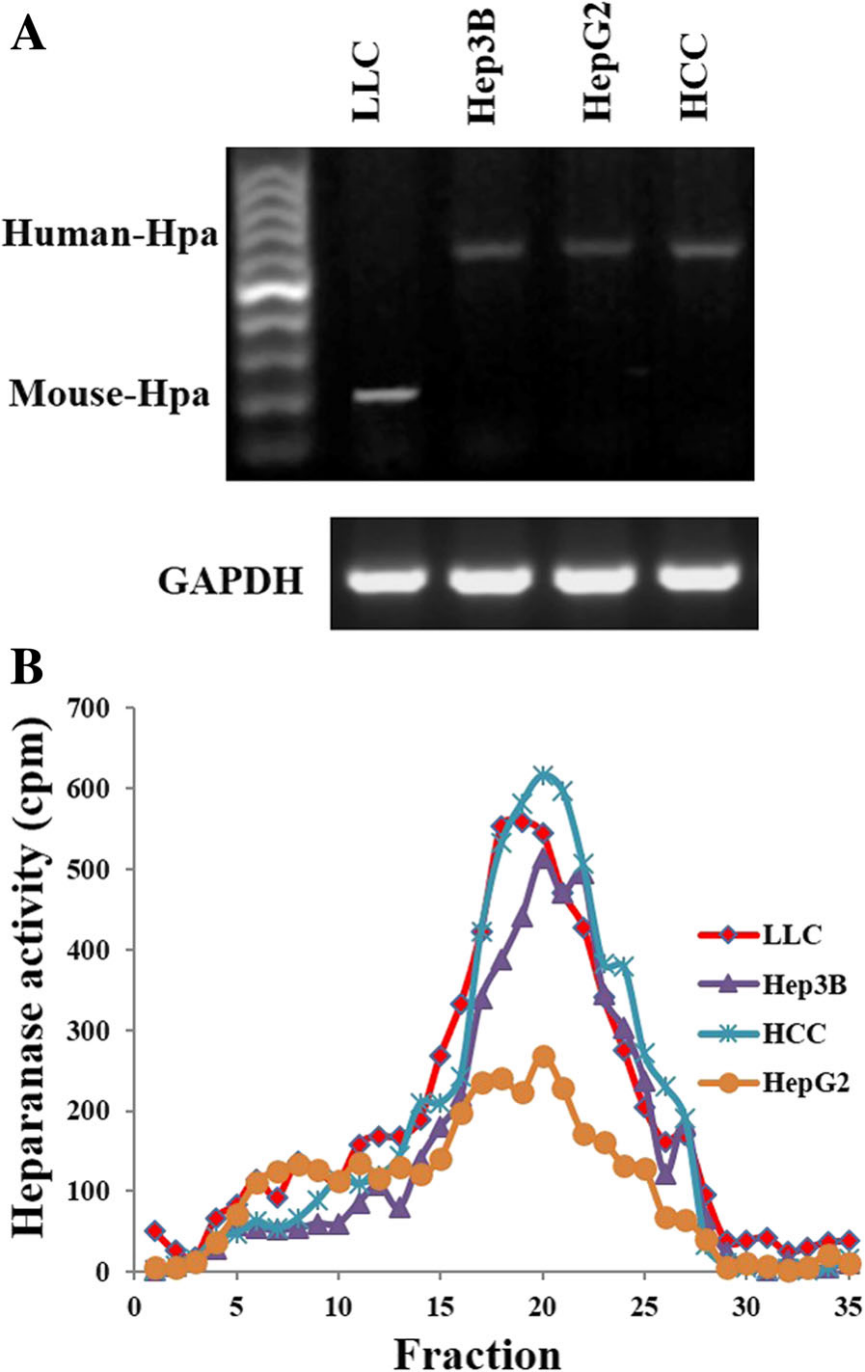
Heparanase expression and activity in various hepatocellular and lung carcinoma cell lines. Mouse Lewis lung carcinoma (LLC), human lung carcinoma (HCC827 = HCC), and human hepatocellular carcinoma (HepG2, Hep3B) cells maintained in culture were subjected to RT-PCR (**a**) and heparanase activity (**b**) assays, as described in ‘Methods’

Heparanase expression (RT-PCR) (Fig. 2a) and enzymatic activity (Fig. 2b) were examined in various hepatocellular carcinoma (human HepG2, Hep3B) and lung carcinoma (human HCC827, mouse LLC) cell lines. A relatively low expression level and enzymatic activity were noted in HepG2 cells as compared to the other cell lines which exhibited moderate-high heparanase enzymatic activity (Fig. 2b). HepG2 human hepatocellular carcinoma and LLC mouse Lewis lung carcinoma cells lines were selected for further experimentation, representing human and mouse cells expressing low (HepG2) and moderate-high (LLC) enzymatic activity, respectively.

### DTP suppresses the proliferation of LLC and HepG2 cells

Given the overexpression of heparanase in hepatocellular and lung carcinoma cancer cell lines, we next analyzed the effect of triazolo-thiadiazoles on LLC (Lewis Lung carcinoma) and HepG2 (hepatocellular carcinoma) cell proliferation using the MTT assay [46–48]. Paclitaxel and DMSO were used as reference drug and vehicle control, respectively. Among the tested triazolo-thiadiazoles, DTP was found to exert an antiproliferative effect with IC_50_ value of 11.9 and 8.3 μM against LLC and HepG2, respectively (Table 1). Thus, structure activity relationship of the lead anticancer agent revealed that phenolic and iodine substituents on the core triazolo-thiadiazole nucleus were found to increase the inhibitory activity towards the proliferation of cancer cells. Notably, the exo-conjugation to the triazolo-thiadiazole core structure also enhances the cytotoxicity. The hydrophobic substituents on the core structure were found to be ineffective against proliferation of cancer cells.

**Table 1:**
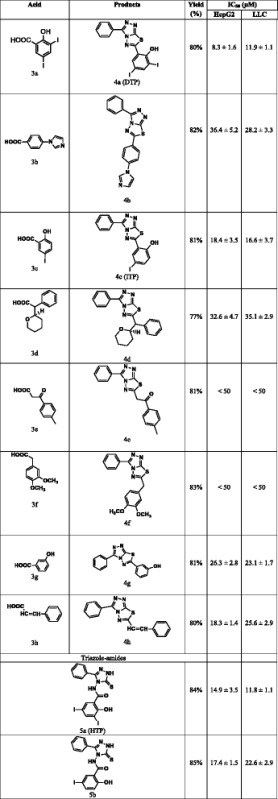
Characterization and anti-proliferative activity of the newly synthesized small molecules that are used for the in vitro heparanase enzyme inhibition studies

### DTP inhibits migration and invasion of LLC cells

**Fig. 3:**
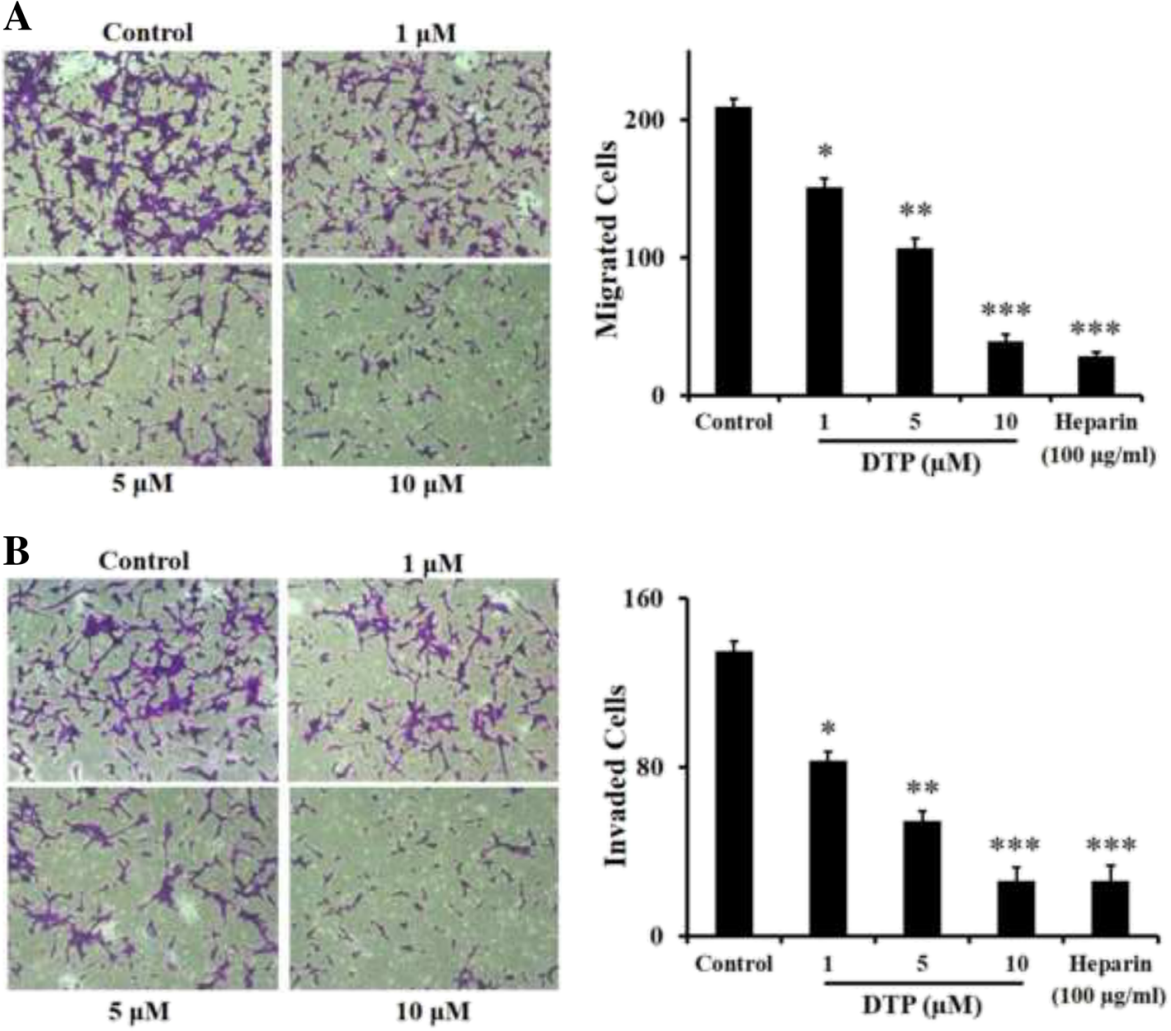
Effect of DTP on LLC cell migration and Invasion. LLC cells were plated on BD BioCoat™ chambers (BD Biosciences) and cell migration (without Matrigel coat) (**a**) and invasion (with Matrigel coat) (**b**) were measured as described in ‘Methods’. The effect of lead compound DTP (1–10 μM) or heparin (100 μg/mL) on cell migration and invasion is demonstrated by representative photomicrographs (magnification: ×10) and the respective bar graphs. Data are represented as mean ± S.E. *P < 0.1; **P < 0.05. ***P < 0.01

**Fig. 4:**
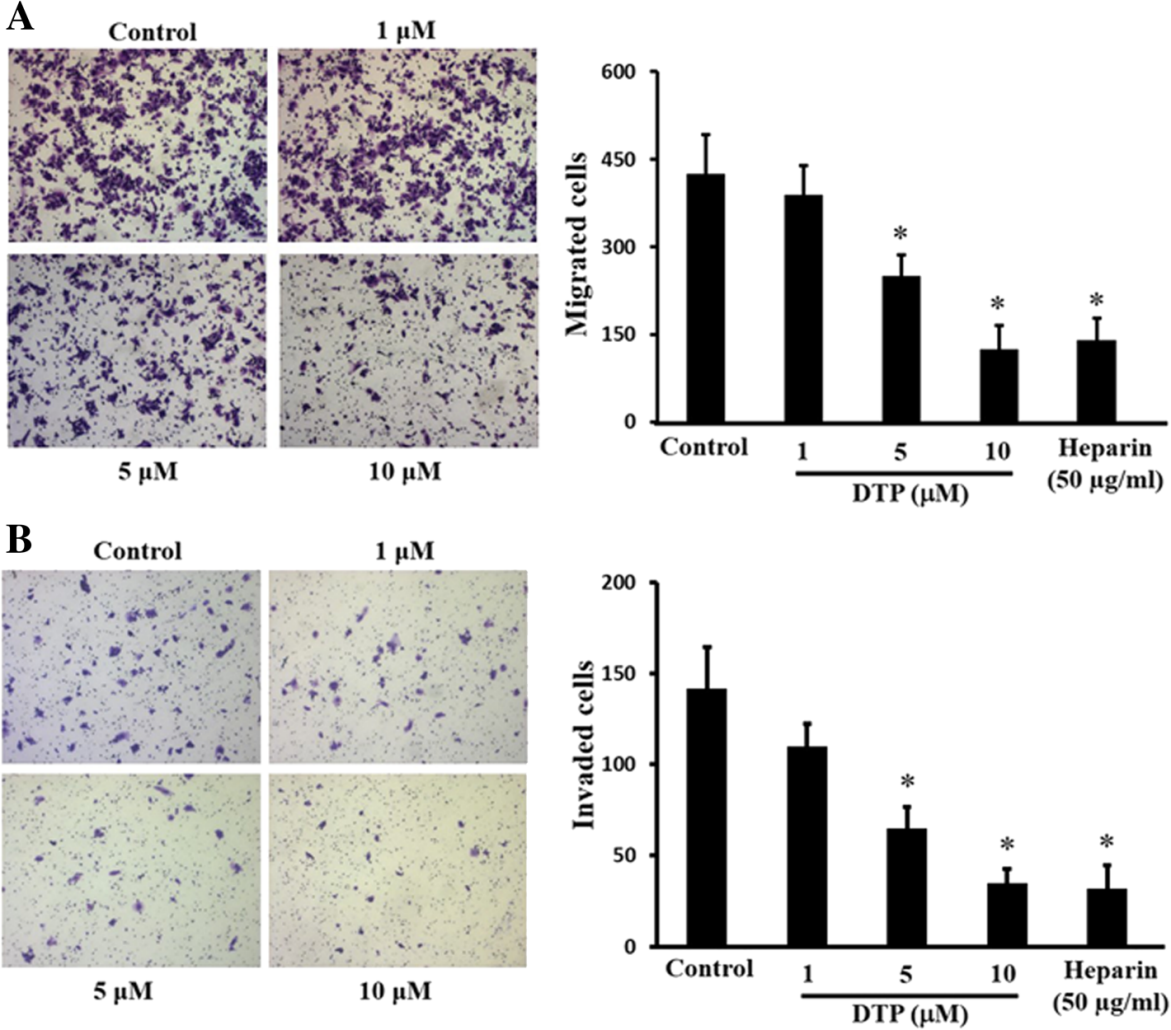
Effect of DTP on HepG2 cell migration and Invasion. HepG2 cells were plated on BD BioCoat™ chambers (BD Biosciences) and cell migration (without Matrigel coat) (**a**) and invasion (with Matrigel coat) (**b**) were measured as described in ‘Methods’. The effect of lead compound DTP (1–10 μM) or heparin (100 μg/mL) on cell migration and invasion is demonstrated by representative photomicrographs (magnification: ×5) and the respective bar graphs. Data are represented as mean ± S.E. *P < 0.05

The involvement of heparanase in cancer metastasis is clearly demonstrated in various types of cancer [9, 14, 32]. We investigated the effect of DTP on LLC and HepG2 cell migration and invasion applying trans-well filters (8 μM pore size) that were either uncoated or coated with Matrigel, respectively. LLC (Fig. 3) and HepG2 (Fig. 4) cells migrated through uncoated filters and invaded through Matrigel in response to stimulation with FBS. DTP significantly suppressed cell migration (Figs. 3a and 4a) and invasion (Figs. 3b and 4b) in a dose dependent manner, yielding nearly 50% inhibition at 5 μM. This effect is likely attributed to inhibition of heparanase enzymatic activity by DTP. Heparin was used as positive control.

### Rationalizing SAR trends via protein-ligand interactions

In order to perform virtual screening, a recently published X-ray crystal structure for human heparanase was obtained from the Protein Data Bank (PDB:5E97; Glycoside Hydrolase ligand structures 1, 1.63 Å resolution) [49]. The structure was loaded into MOE [50] and corrected using the Structure Preparation tool before running Protonate 3D. The Site Finder tool identified the active site containing Glu-343 and Glu-225 that were identified as the catalytic nucleophile and acid-base of Heparanase [45, 49]. Compound structures were loaded into MOE and energy minimised before carrying out rigid receptor docking (triangle matcher, London dG Forcefield refinement, GBVI/WSA dG rescoring).

**Fig. 5:**
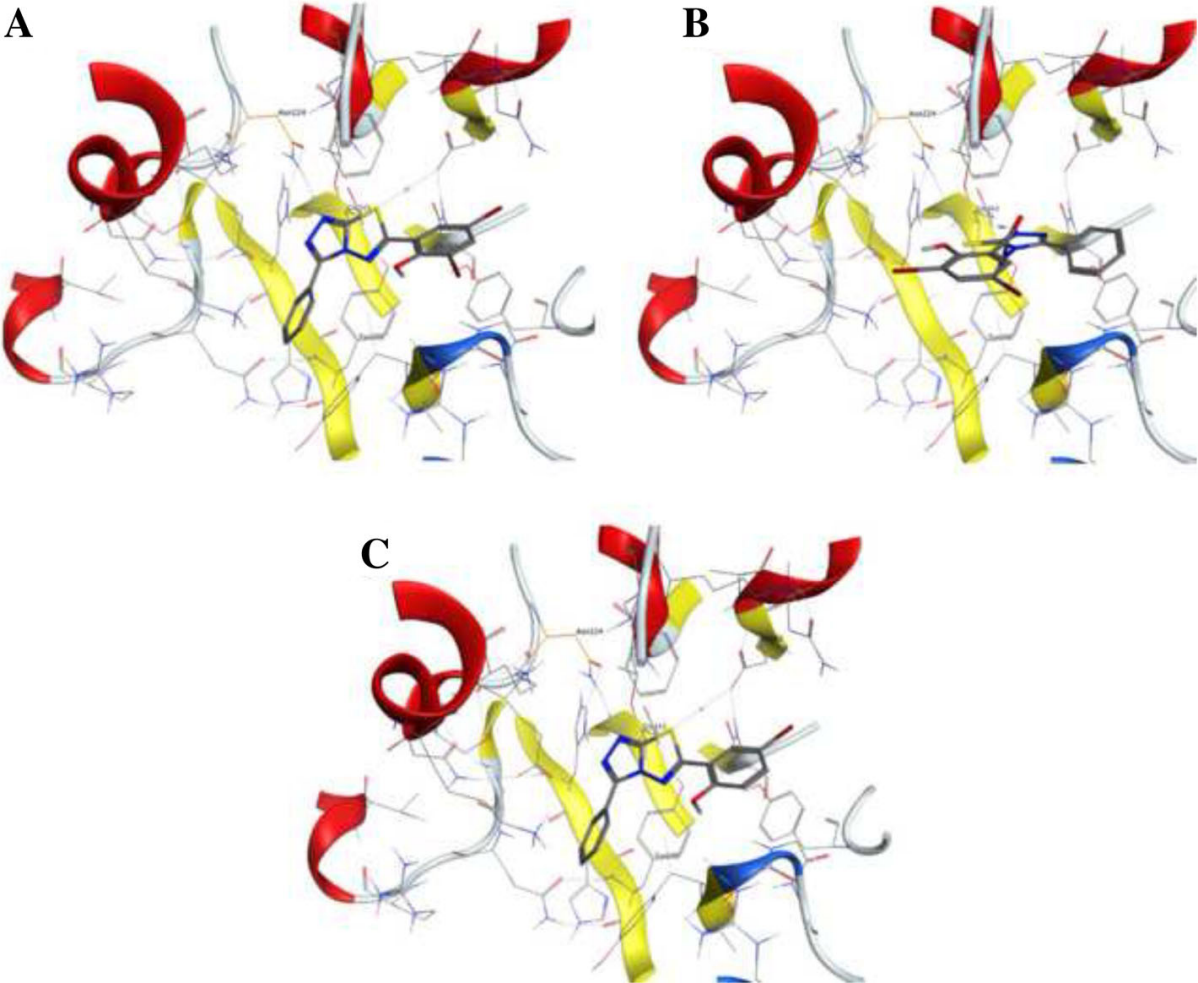
Selected docked poses for active compounds DTP, HTP and ITP (**a**, **b** and **c**, respectively), showing similar active site interaction modes. DTP and ITP are shown to interact with both Asn-224 and Asp-62 via the triazolo-thiadiazole backbone, and HTP is shown to interact with Asn-224 and the active site acid-base Glu-343

The 52 docked poses that included the three active compounds DTP, HTP, and ITP did not appear to explain the experimentally observed trend in SAR. However, docking results revealed a similar interaction pattern between active compounds ITP and DTP, with poses that interact favourably with both Asn-224 and Asp-62 due to the triazolo-thiadiazole backbone (Fig. 5). For compound HTP, this interaction profile was found to be slightly less favourable, interacting instead with Asn-224 and the active site acid-base Glu-343.

Although these compounds do not appear to be more favourable than the other docked compunds, the presence of iodine substituents found on all hit compounds may preferentially lower the phenols’ pKA sufficiently to allow for deprotonation of the ligands in protein environment.

## Discussion

Human heparanase is an endoglucuronidase that cleaves heparan sulfate chains thereby regulating multiple biological activities that together enhance tumor growth, metastasis and angiogenesis [7–10, 14, 32]. Heparanase is expressed by most types of cancer and has emerged as a valid target for anti-cancer therapy [8, 15]. Heparanase represents a druggable target because: (i) there is only a single enzymatically active heparanase expressed in humans, (ii) the enzyme is present in low levels in normal tissues but dramatically elevated in tumors where it is associated with poor prognosis and reduced postoperative survival time, and (iii) heparanase deficient mice appear normal [51]. Thus, properly designed heparanase inhibitors will likely have few, if any, negative side effects. Development of heparanase inhibitors has focused predominantly on carbohydrate-based compounds with heparin-like properties [8, 15, 44]. These compounds bind to the heparin-binding domains that flank the enzyme active site of heparanase thereby inhibiting cleavage of heparan sulfate. Four different heparin mimics are currently in clinical trials in human cancer patients. However all of these mimics have the disadvantage that they are not specific for heparanase and likely interact with different heparin-binding proteins with unknown consequences and off target effects [8, 15]. Therefore, even if they prove efficacious in patients it will be impossible to attribute their effect solely to heparanase inhibition. In addition three of the four mimics are heterogeneous in their structure adding further to their uncertainty as viable drugs for use in humans [8]. A number of heparanase-inhibiting small molecules were reported [8, 16, 22], but none entered clinical testing.

Heparanase expressed in cancer cells and cells of the tumor microenvironment provides a most appropriate therapeutic molecular target and could serve a decisive role in cancer regime. In addition to remodeling of ECM, human heparanase regulates multiple signaling cascades involved in tumor cell survival, angiogenesis and metastasis [7, 8, 14, 15, 44, 52]. The positive correlation of heparanase with progression of malignancies makes it an attractive target in the treatment of cancer. It is hoped that our identification of a lead molecule and the recently resolved crystal structure of the heparanase protein [49] will accelerate rational design of heparanase-inhibiting small molecules endowed with considerably improved binding affinity, specificity, pharmacokinetics and efficacy in xenograft cancer models. Selected molecules exerting little or no side effects will then be examined for oral availability and anti cancer effect in combination with currently available treatments, applying patient derived xenograft models and, at a later stage, animal models of other diseases shown to be causally related to heparanase [53–58].

## Conclusions

In a search for small molecule inhibitors that can interfere with the catalytic activity of human heparanase, we report the synthesis and biological evaluation of a library of synthetic small molecules and identification of triazolo-thiadiazole derivative as a potent inhibitor of human heparanase. The identified lead structure displayed antiproliferative activity and suppressed the migration and invasion of cancer cells in correlation with inhibition of heparanase enzymatic activity. Further development of this novel class of heparanase inhibitors and optimization to maximize their affinity, pharmacokinetics and oral availability will provide a unique opportunity for development of innovative anti-cancer therapeutics. Moreover, because heparanase helps drive the progression of other diseases (e.g., diabetes, diabetic nephropathy, arthritis, colitis, sepsis, atherosclerosis) [53–58], these drugs hold potential to impact public health.

## Additional file

Additional file 1: Table S1. Optimisation of mol% of SCe catalyst, and selection of medium for cyclization reaction. To optimize the reaction conditions for the synthesis of novel 1,2,4-triazolo-1,3,4-thiadiazoles, the reaction was performed in combination of 4-amino-5-phenyl-4 h-1,2,4-triazole-3-thiol and 3-oxo-3-(p-tolyl)propanoic acid as a model reaction in different concentrations of SCe and solvent. The optimal system for cyclization was 20 mol% of SCe in DMF. **Table S2.** Evaluation of the reuse of SCe for cyclization reaction. The recyclability of the SCe system was evaluated by employing 4-amino-5-phenyl-4 h-1,2,4-triazole-3-thiol with 3-oxo-3-(p-tolyl)propanoic acid to yield 2-(3-Phenyl-[1, 2, 4]triazolo[3,4-b][1, 3, 4]thiadiazol-6yl)-1-p-tolylethanone. The catalyst was removed by filtration after each run and thoroughly washed with acetone, dried and activated at 823 K and taken for the next cycle. There was a significant reduction in the yield of the product after the second run using SCe. **Figure S1.** Spectral data. Scanned copy of ^1^H NMR, ^13^C NMR, and mass spectra of the indicated compounds. (DOCX 4202 kb)

## Abbreviations

BSA: Bovine serum albumin
DMEM: Dulbecco’s Modified Eagle Medium
DTP: 2,4-Diiodo-6-(3-phenyl-[1, 2, 4]triazolo[3,4-b][1, 3, 4]thiadiazol-6yl)phenol
ECM: Extracellular matrix
HGF: Hepatocyte growth factor
HS: Heparan Sulfate
HSPG: heparan sulphate proteolglycan
HTP: 2-Hydroxy-3,5-diiodo-N-(3-phenyl-5-thioxo-1H-1,2,4-triazol-4(5H)-yl)benzamide
ITP: 4-Iodo-2-(3-phenyl-[1, 2, 4]triazolo[3,4b][1, 3, 4]thiadiazol-6-yl)phenol.
LLC: Lewis lung carcinoma
MOE: Molecular operating environment
MTT: 3-(4,5-dimethylthiazol-2-yl)-2,5-diphenyltetrazolium bromide
NMR: Nuclear magnetic resonance
OD: Optical density
SAR: Structural activity relationship
SCe: sulphated ceria
TLC: Thin layer chromatography
VEGF: Vascular endothelial growth factor
