# Supplementary material for: Identification of Novel Class of Triazolo-Thiadiazoles as Potent Inhibitors of Human Heparanase and their Anticancer Activity
Source: BMC Cancer. 2017 Mar 31;17:235. doi: 10.1186/s12885-017-3214-8 (PMC5374561; doi:10.1186/s12885-017-3214-8)

**Additional file 1**

**Identification of Novel Class of Triazolo-Thiadiazoles as Potent Inhibitors of Human Heparanase and their Anticancer Activity**

**Table S1:** Optimisation of SCe catalyst and medium for cyclization.

| Entry | Catalyst | Solvent | Time  (h) | Yield ( %) |
| --- | --- | --- | --- | --- |
| 1 | SCe (10mol%) | Ethanol | 15 | NR |
| 2 | SCe (10mol%) | DMF | 15 | NR |
| 3 | SCe (10mol%) | 1,4 dioxane | 15 | NR |
| 4 | SCe (18mol%) | Ethanol | 15 | NR |
| 5 | SCe (18mol%) | DMF | 15 | 65^b^ |
| 6 | SCe (18mol%) | 1,4 dioxane | 15 | 43 |
| 7 | SCe (20mol%) | Ethanol | 15 | NR |
| 8 | SCe (20mol%) | DMF | 10 | 81^b^ |
| 9 | SCe (20mol%) | 1,4 dioxane | 15 | 60^b^ |
| Conditions: 4-amino-5-phenyl-4h-1,2,4-triazole-3-thiol (1mmol): 3-oxo-3-(p-tolyl)propanoic acid (1mmol):POCl_3_(0.1mmol); Solvent 10mL per mmol substrate, reflux . ^b^ Isolated yield. | | | | |

**Table S2:** Evaluation of the reuse of SCe for cyclization.

| Runs^a^ | 1 | 2 | 3 | 4 |
| --- | --- | --- | --- | --- |
| yield^b^ | 79 | 72 | 61 | 52 |
| ^a^ Conditions: 4-amino-5-phenyl-4h-1,2,4-triazole-3-thiol (1mmol): 3-oxo-3-(p-tolyl)propanoic acid (1mmol): SCe (20mol%) :POCl_3_(0.1mmol); DMF 10mL per mmol substrate, reflux; 10hrs . ^b^ Isolated yield. | | | | |

**Figure S1: Spectral data**

4a

***2,4-Diiodo-6-(3-phenyl-[1,2,4]triazolo[3,4-b][1,3,4]thiadiazol-6yl)phenol***

**
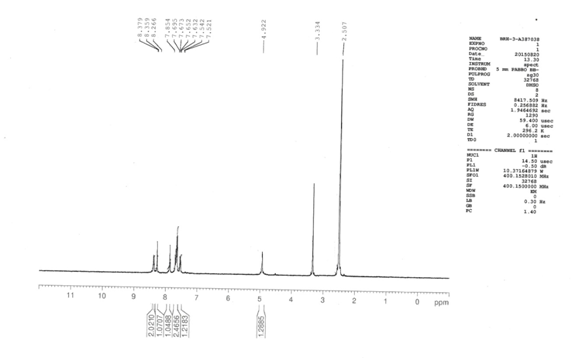
**

**
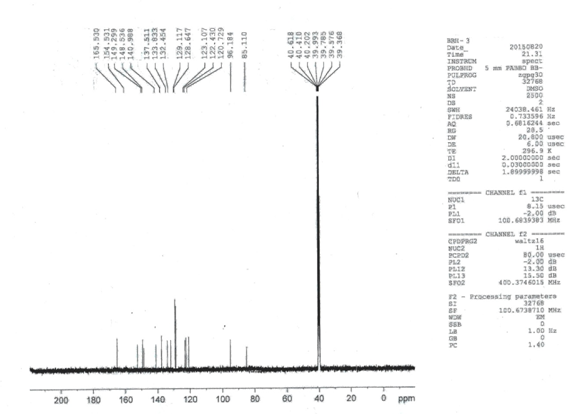
**

**
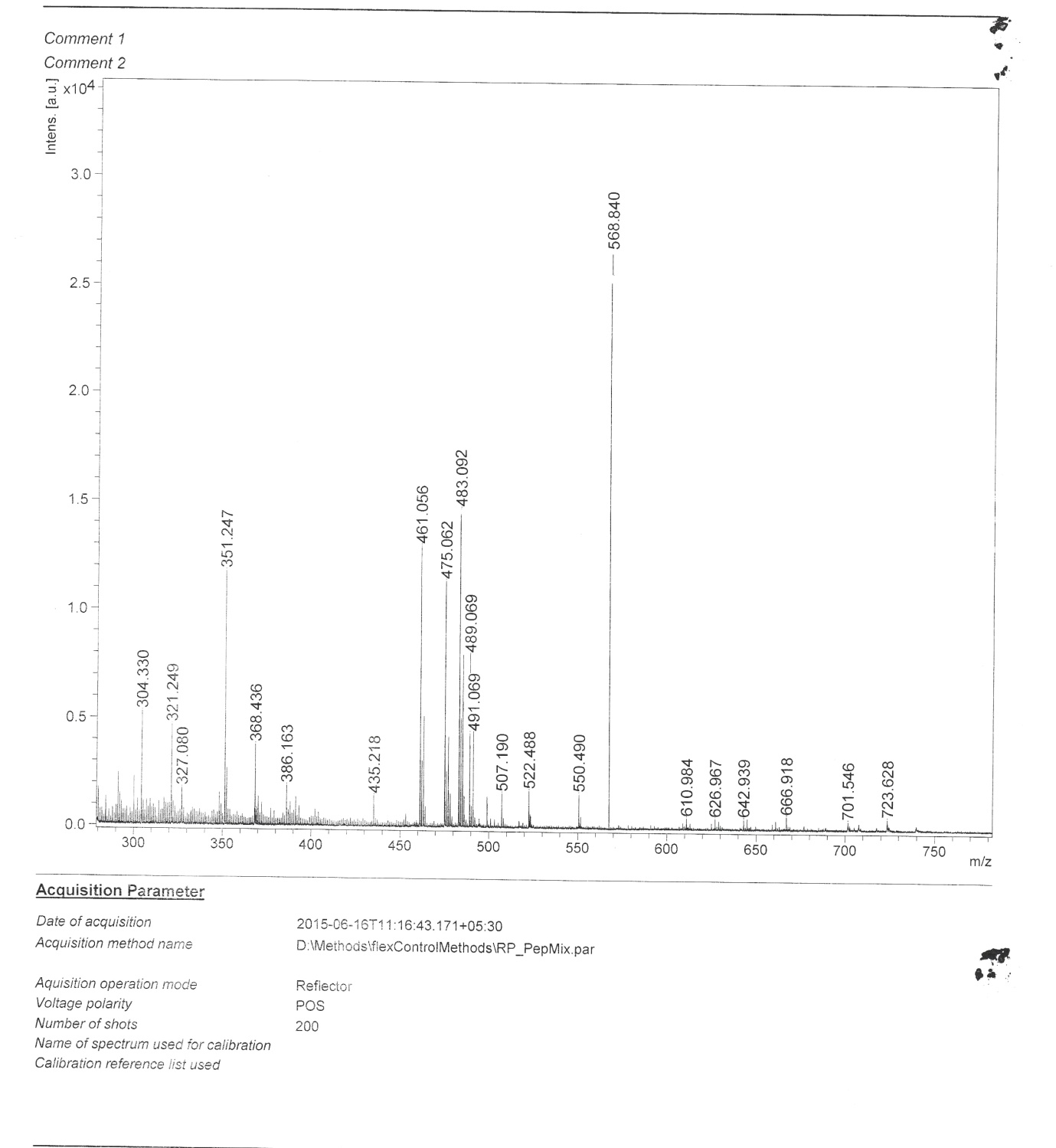
**

(4b)

***6-(4-(1H-imidazol-1-yl)phenyl)-3-phenyl-[1,2,4]triazolo[3,4-b][1,3,4]thiadiazole (4b)***


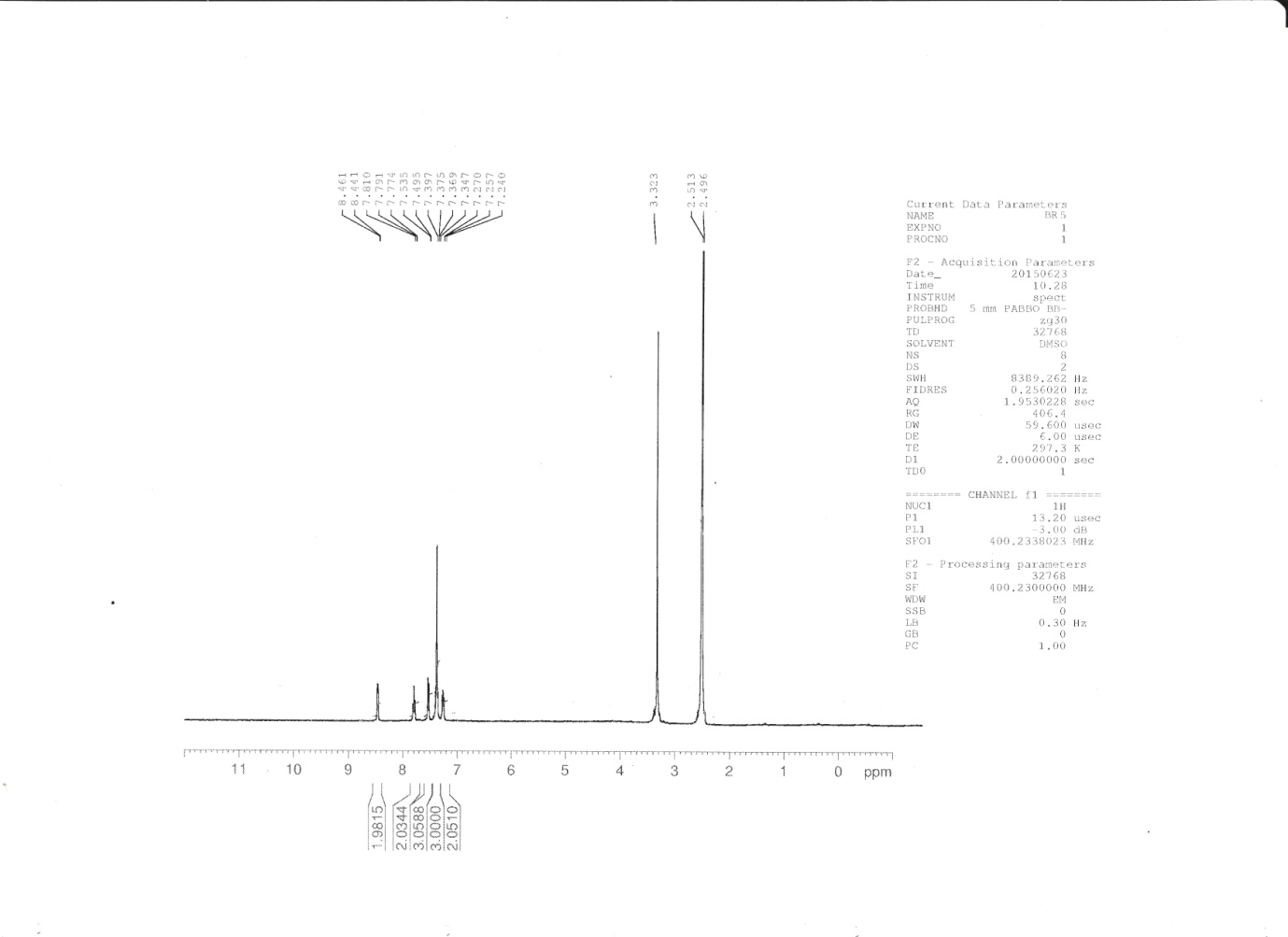


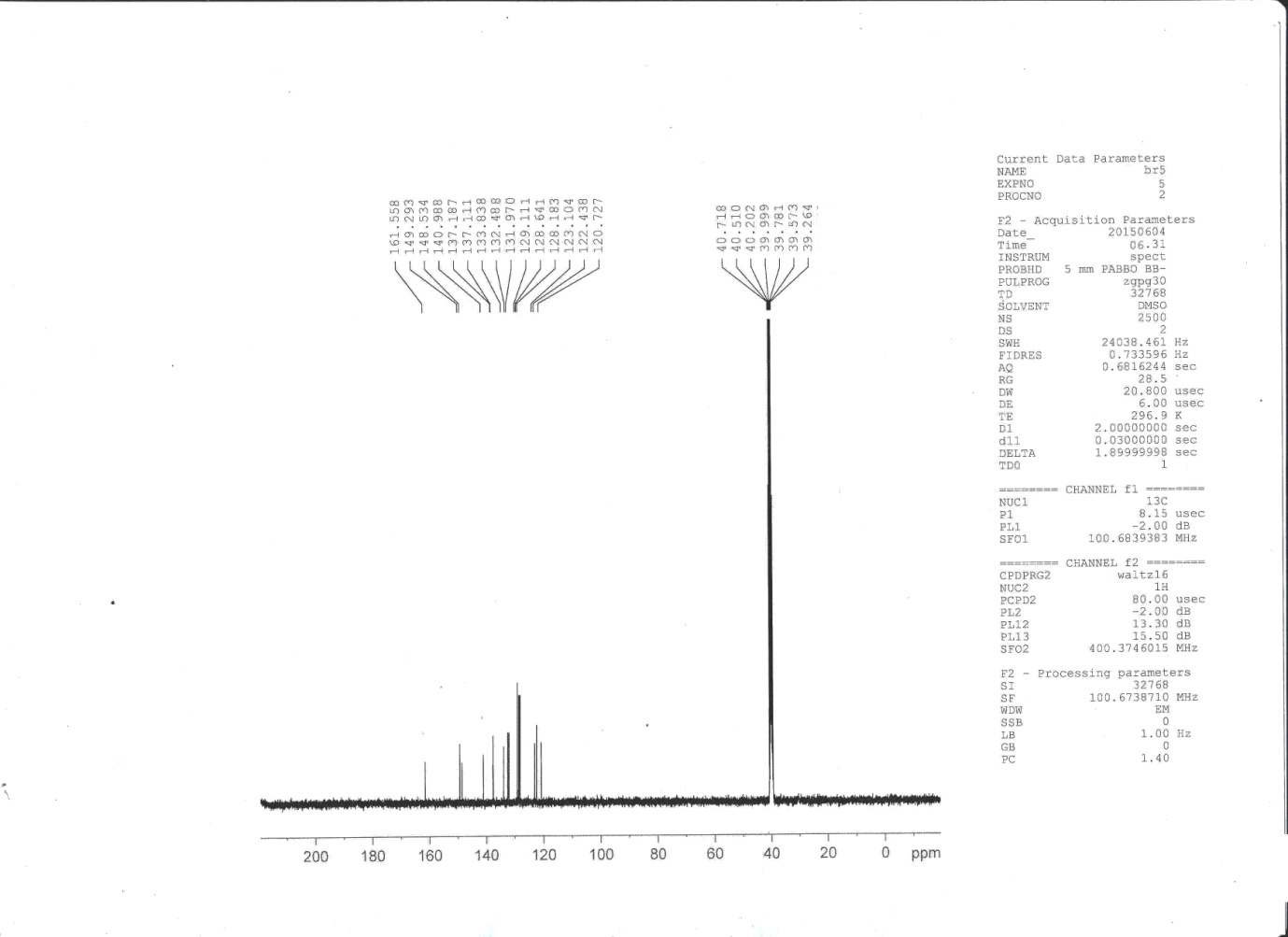


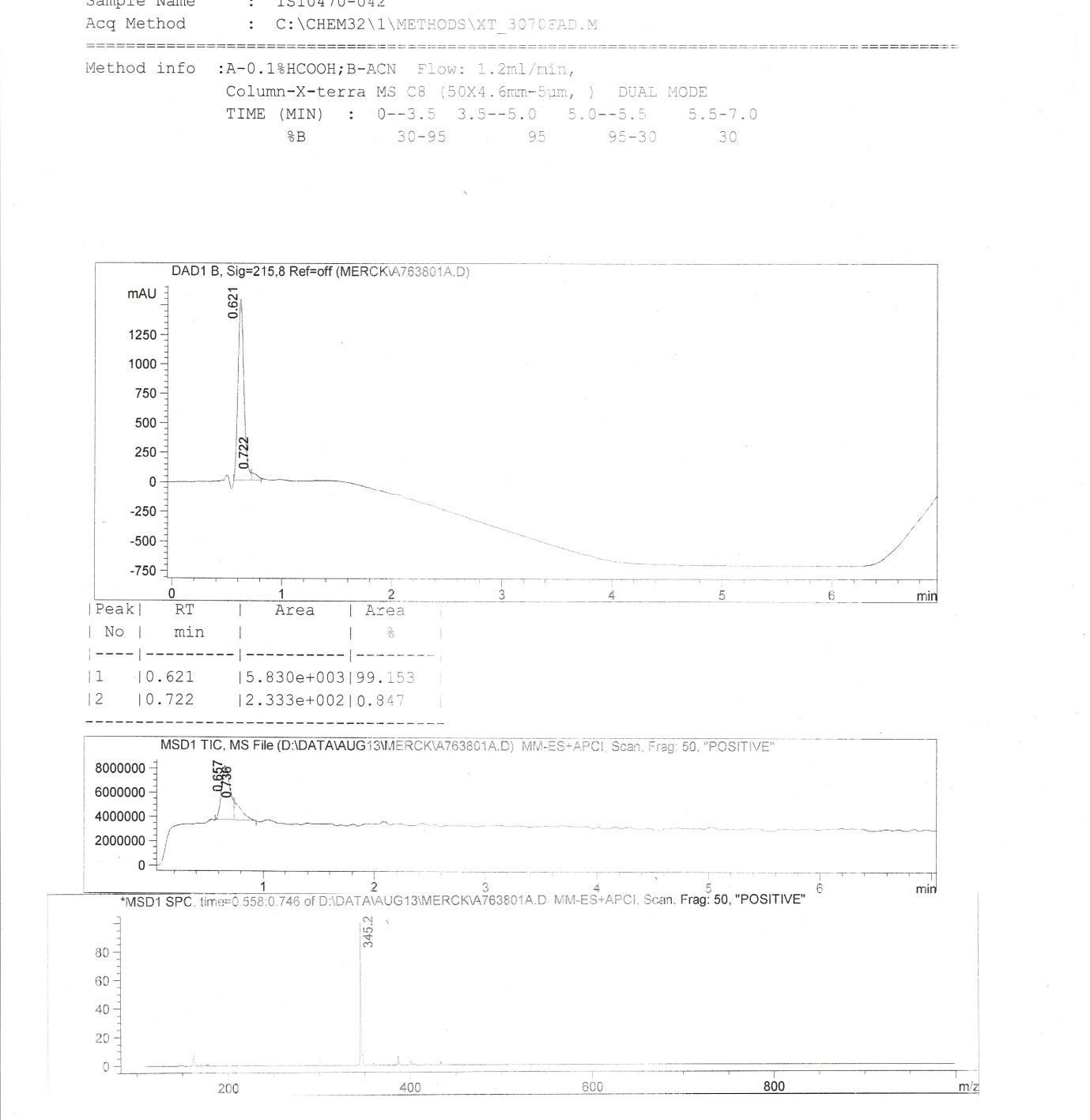

(4c)

***4-iodo-2-(3-phenyl-[1,2,4]triazolo[3,4-b][1,3,4]thiadiazol-6-yl)phenol (4c)***


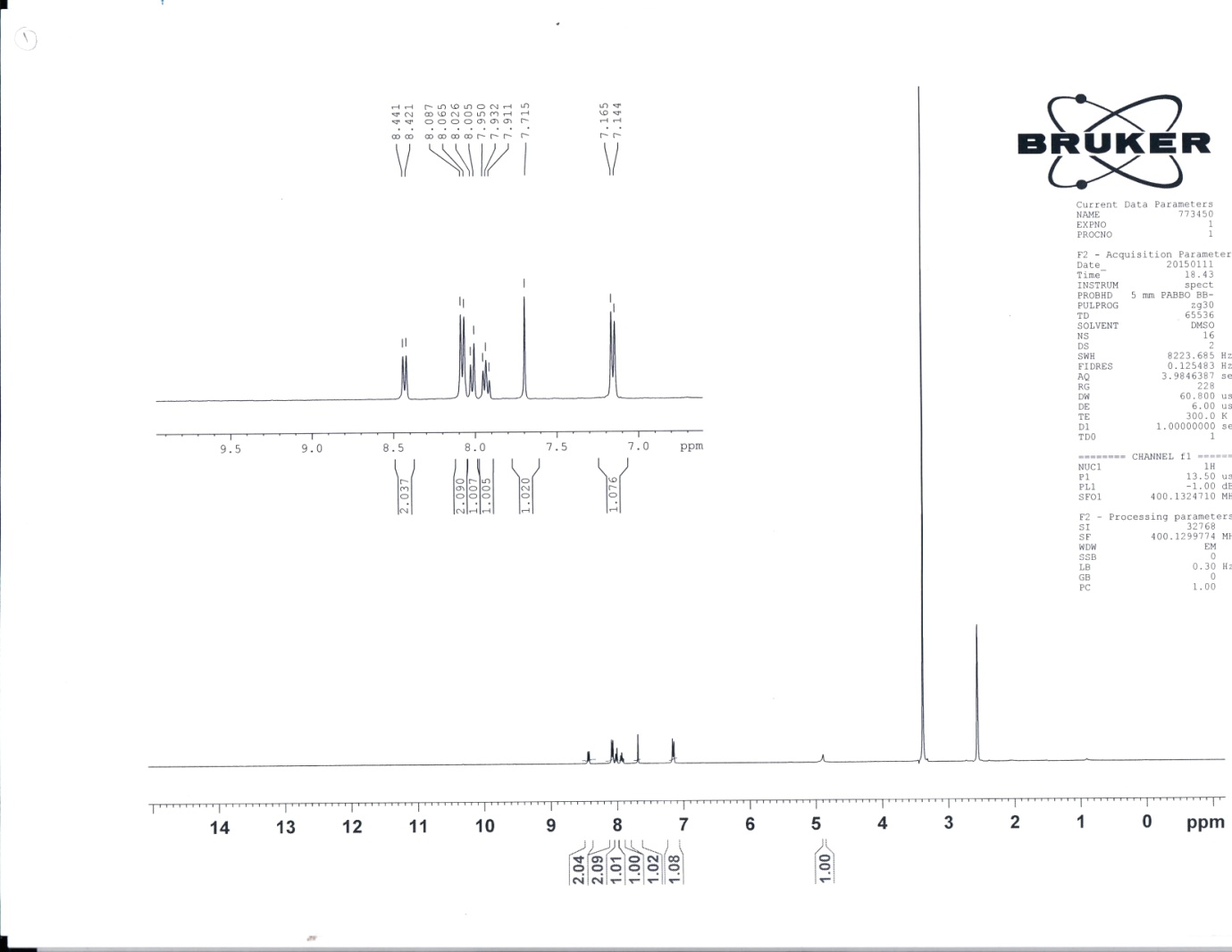


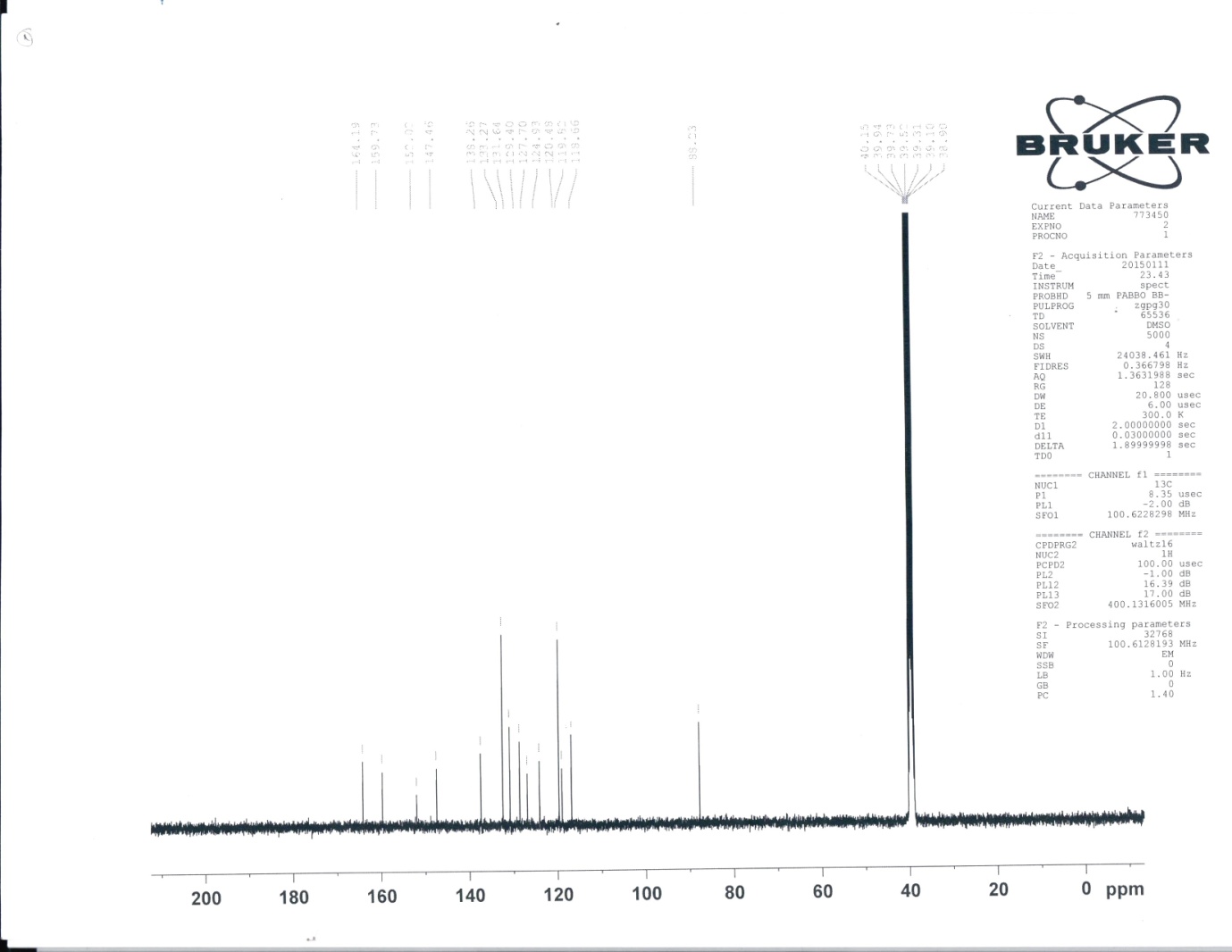


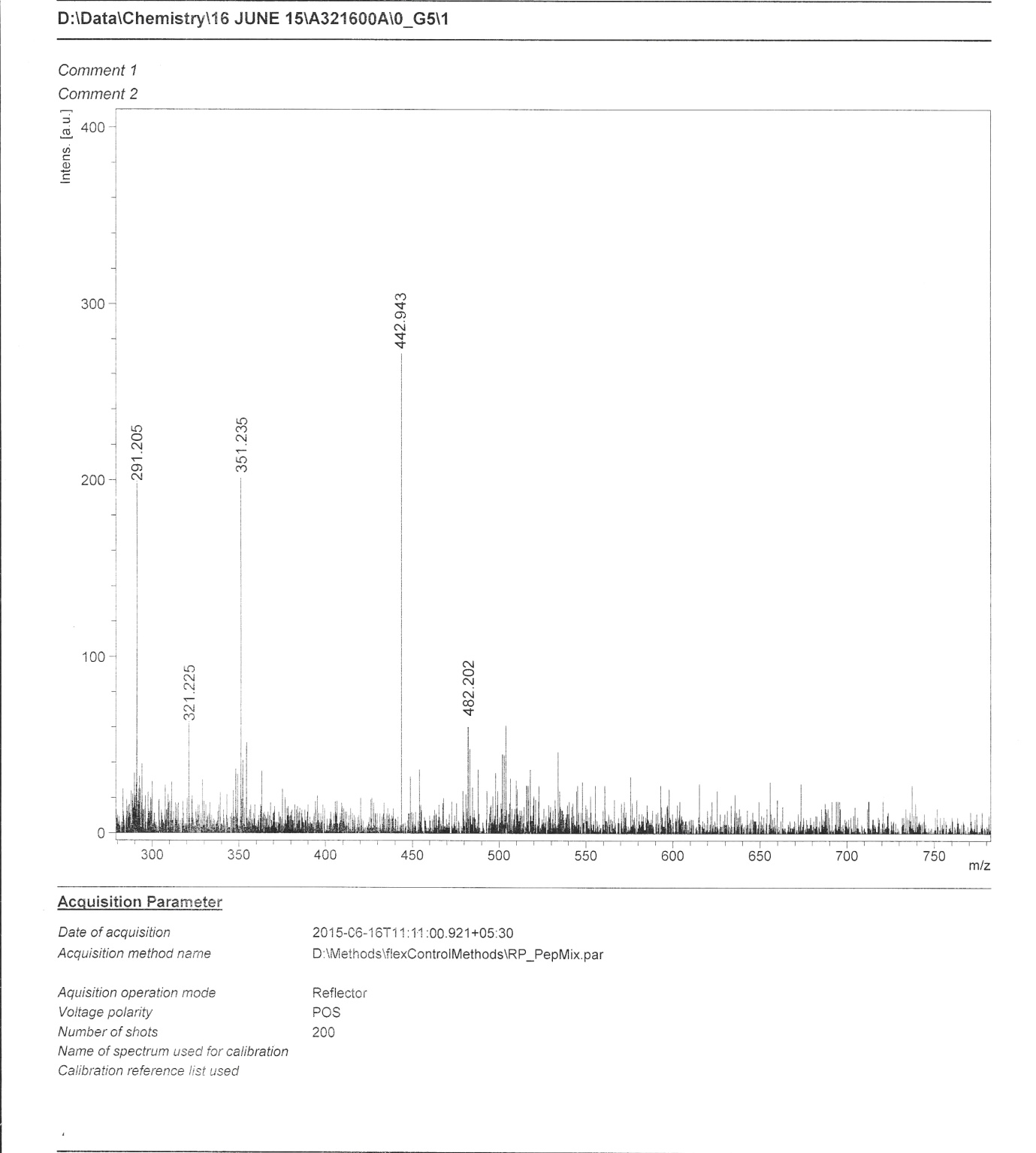

(4d)

***6-(((R)-tetrahydro-2H-pyran-2-yl)(phenyl)methyl)-3-phenyl-[1,2,4]triazolo[3,4-b][1,3,4]thiadiazole (4d)***


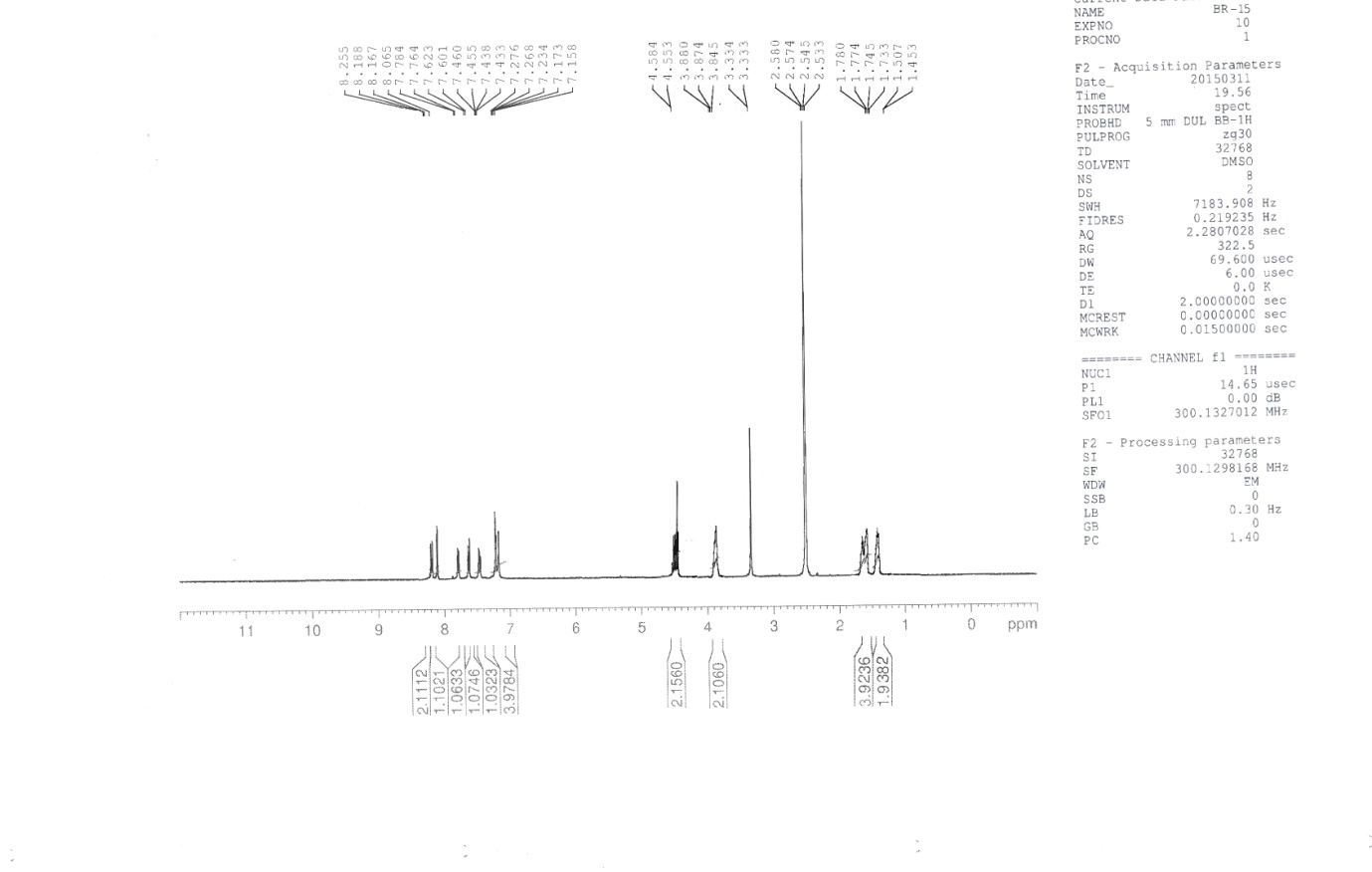


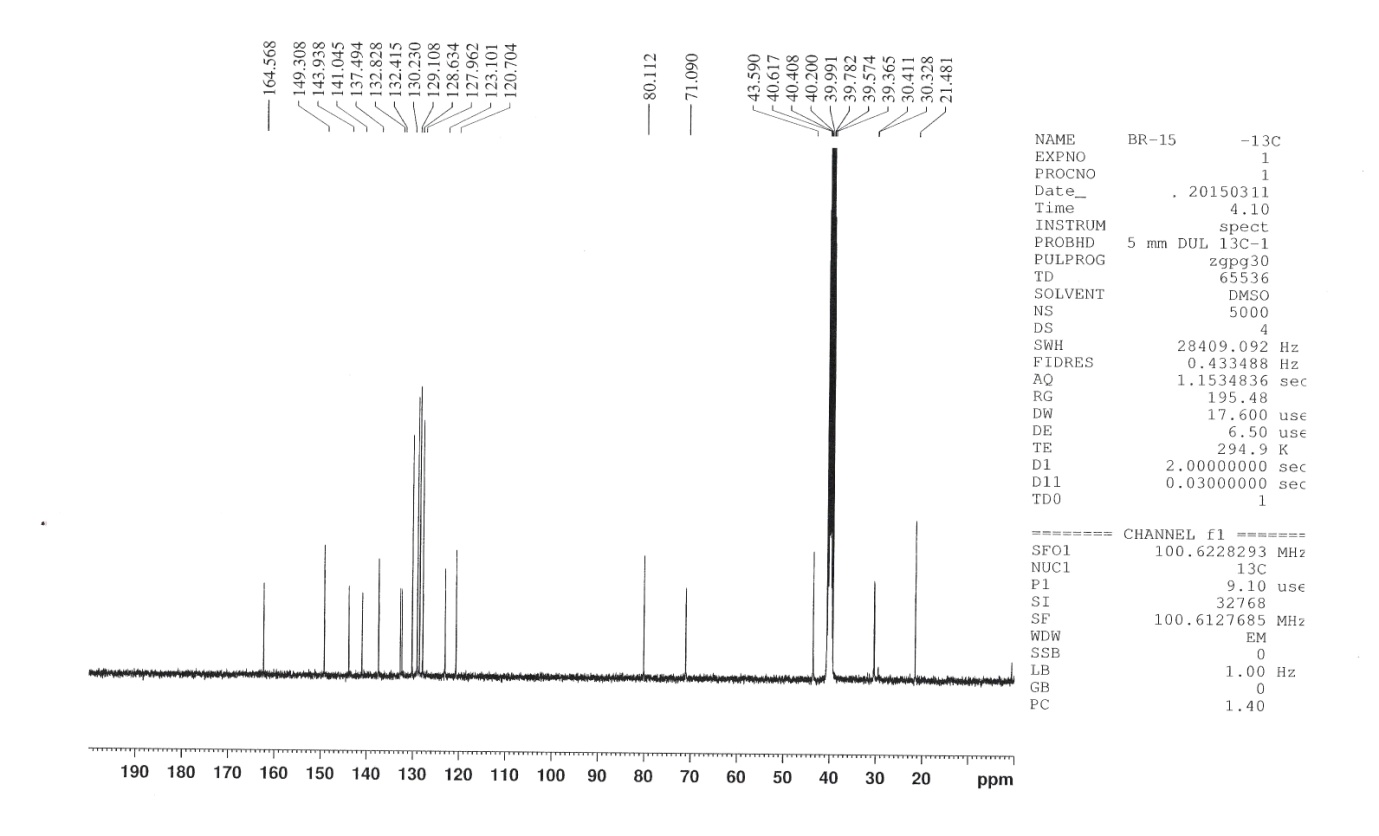


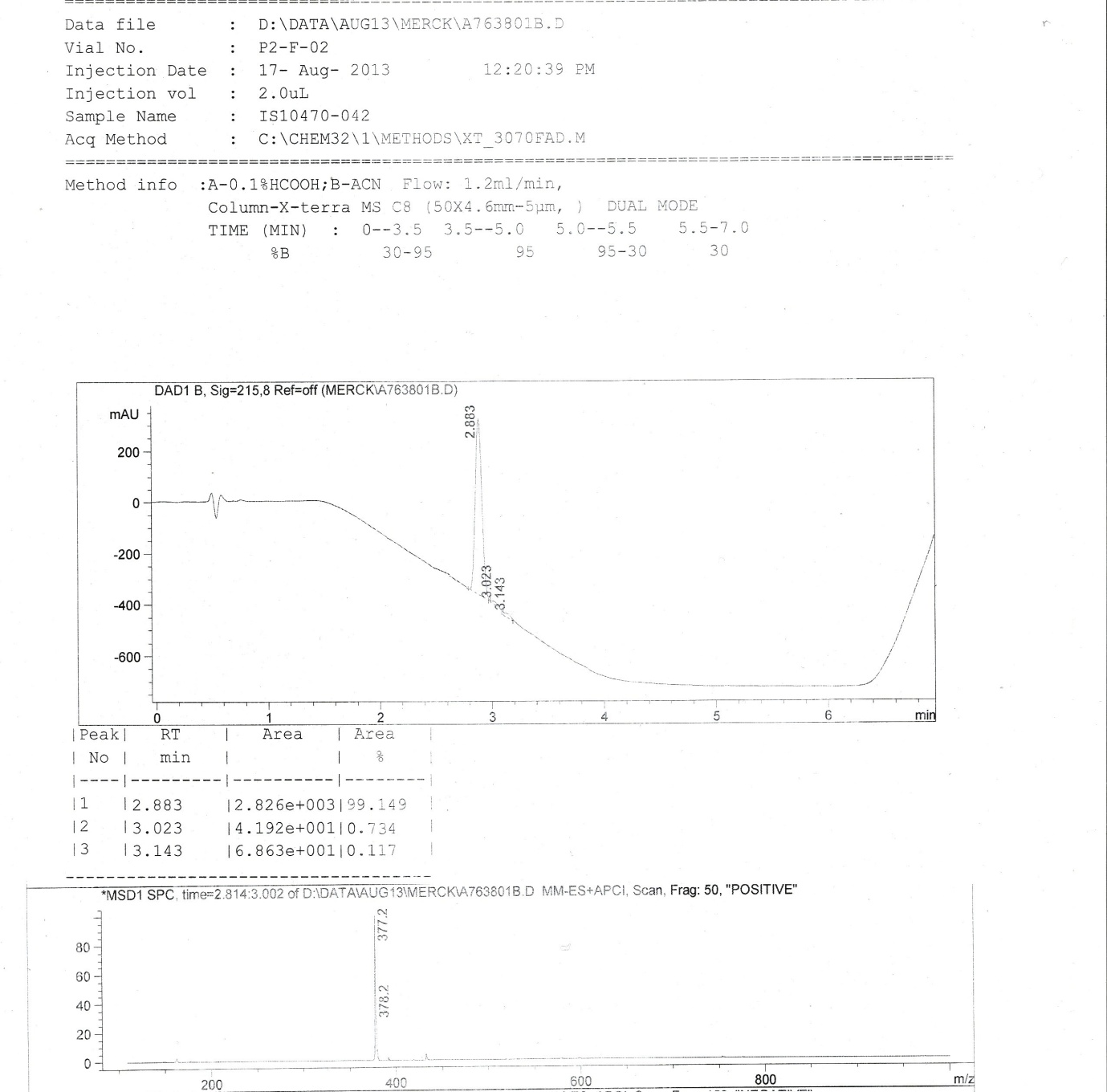

(4e)

***2-(3-phenyl-[1,2,4]triazolo[3,4-b][1,3,4]thiadiazol-6yl)-1-p-tolylethanone (4e)***


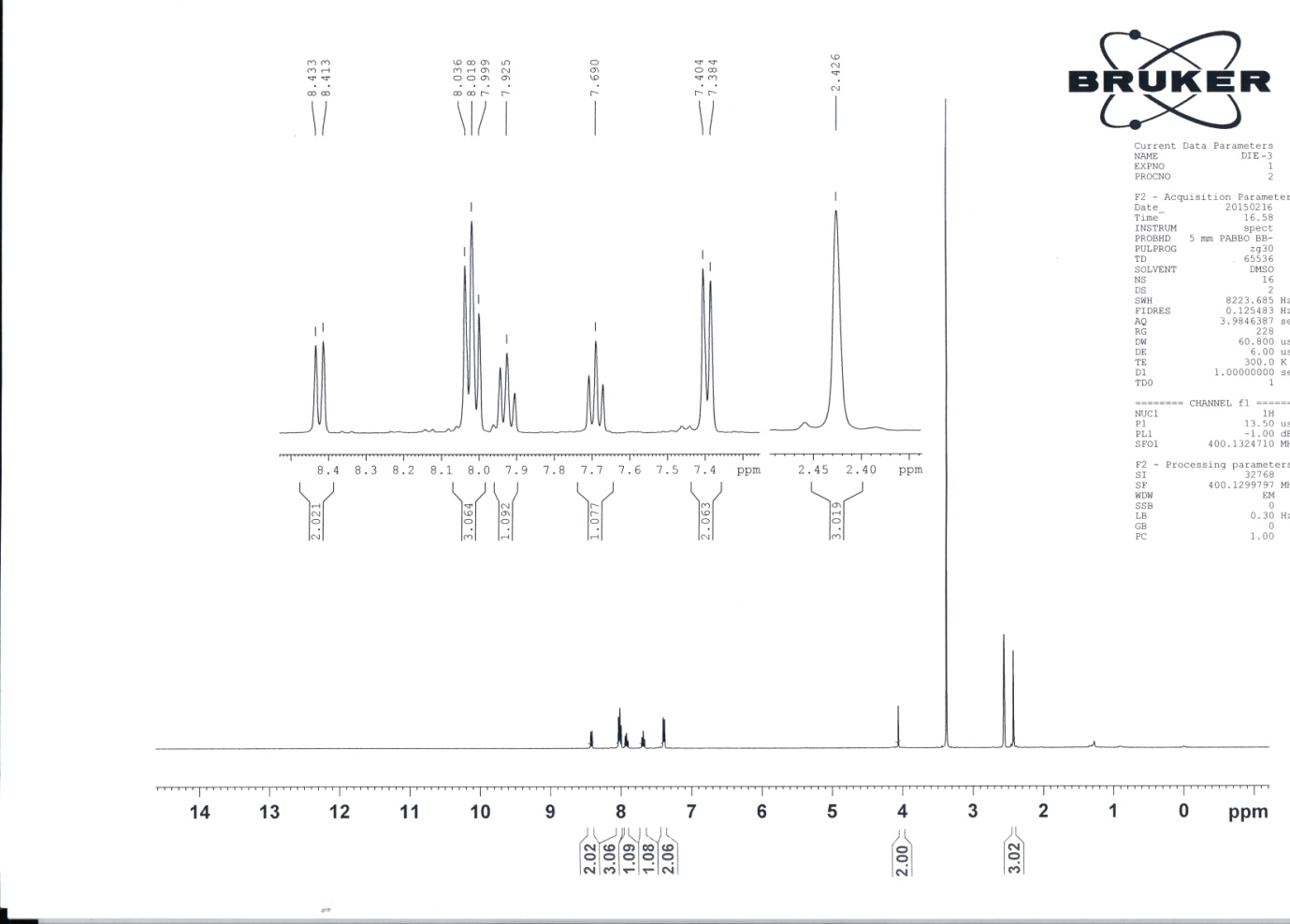


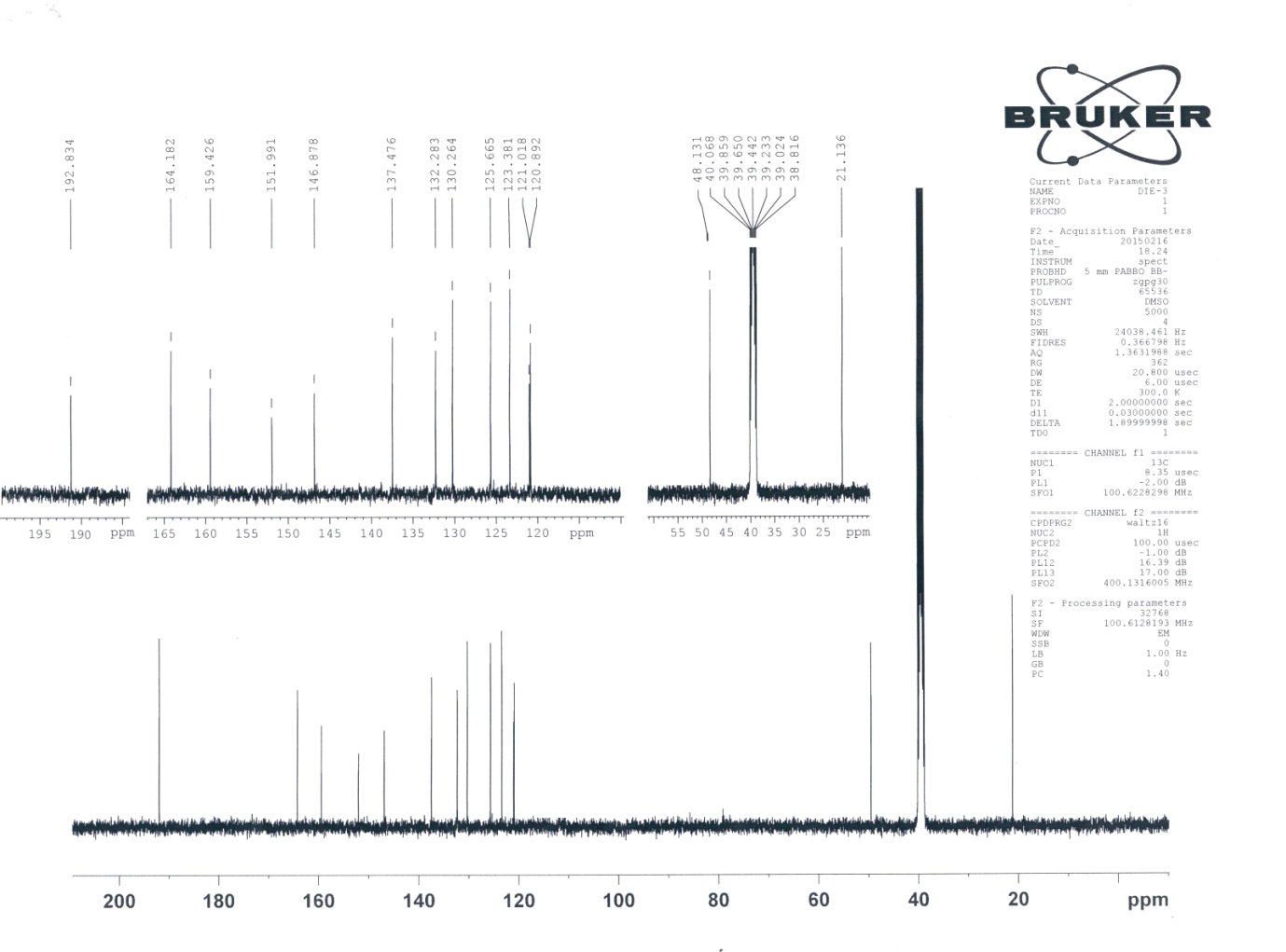


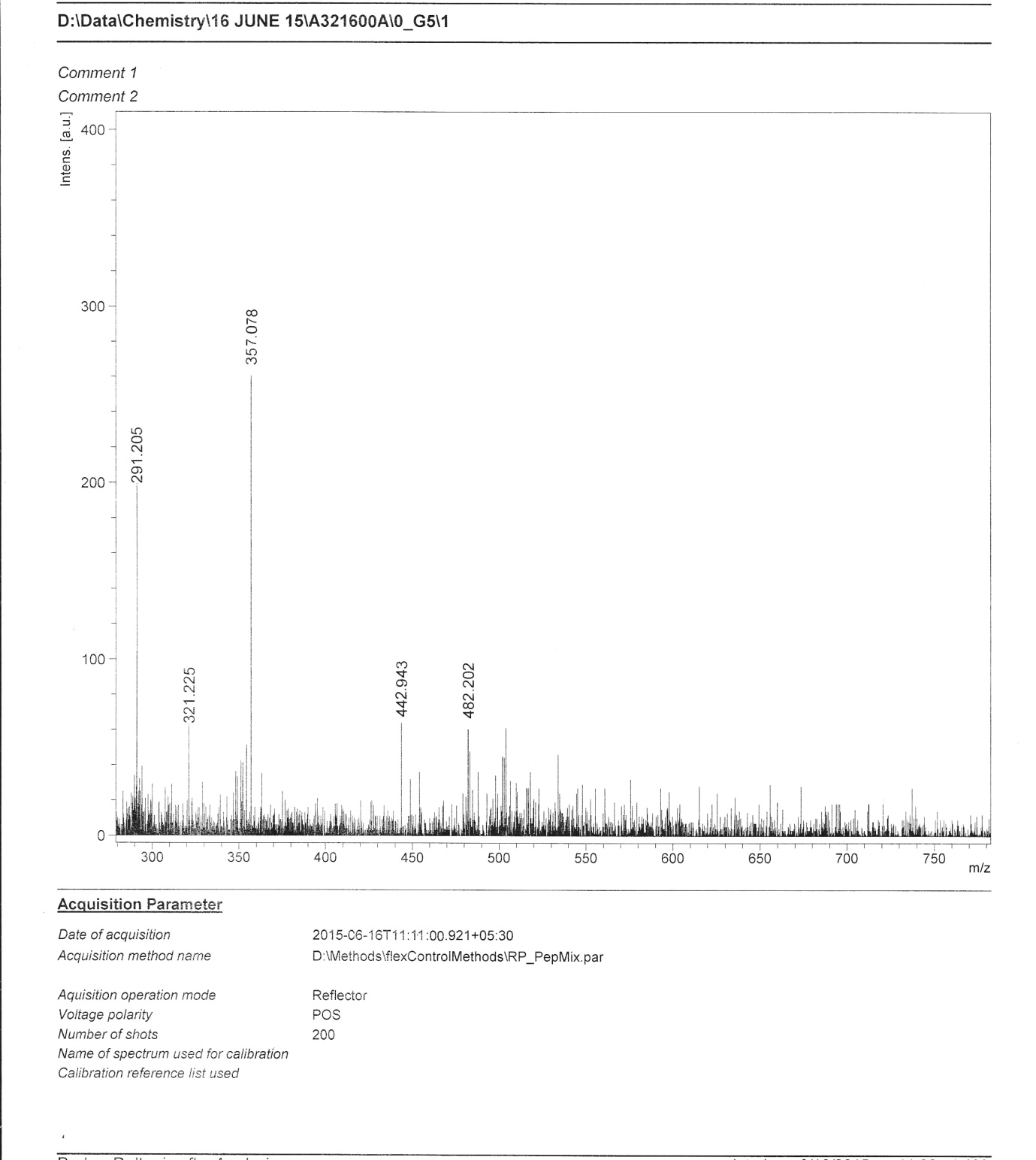

(4f)

***6-(3-4-dimethoxybenzyl)-3-phenyl-[1,2,4]triazolo[3,4-b][1,3,4]thiadiazole (4f)***


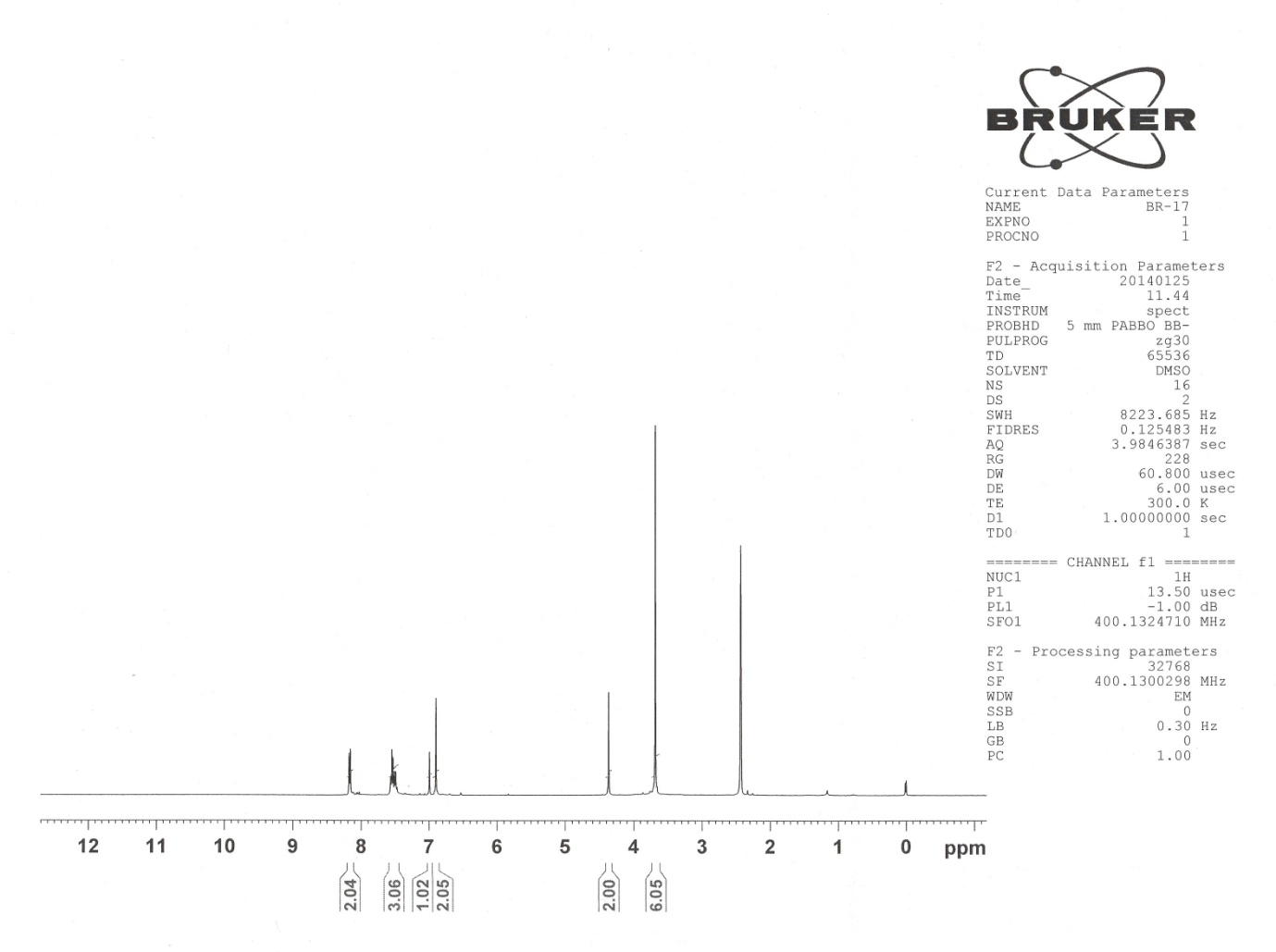


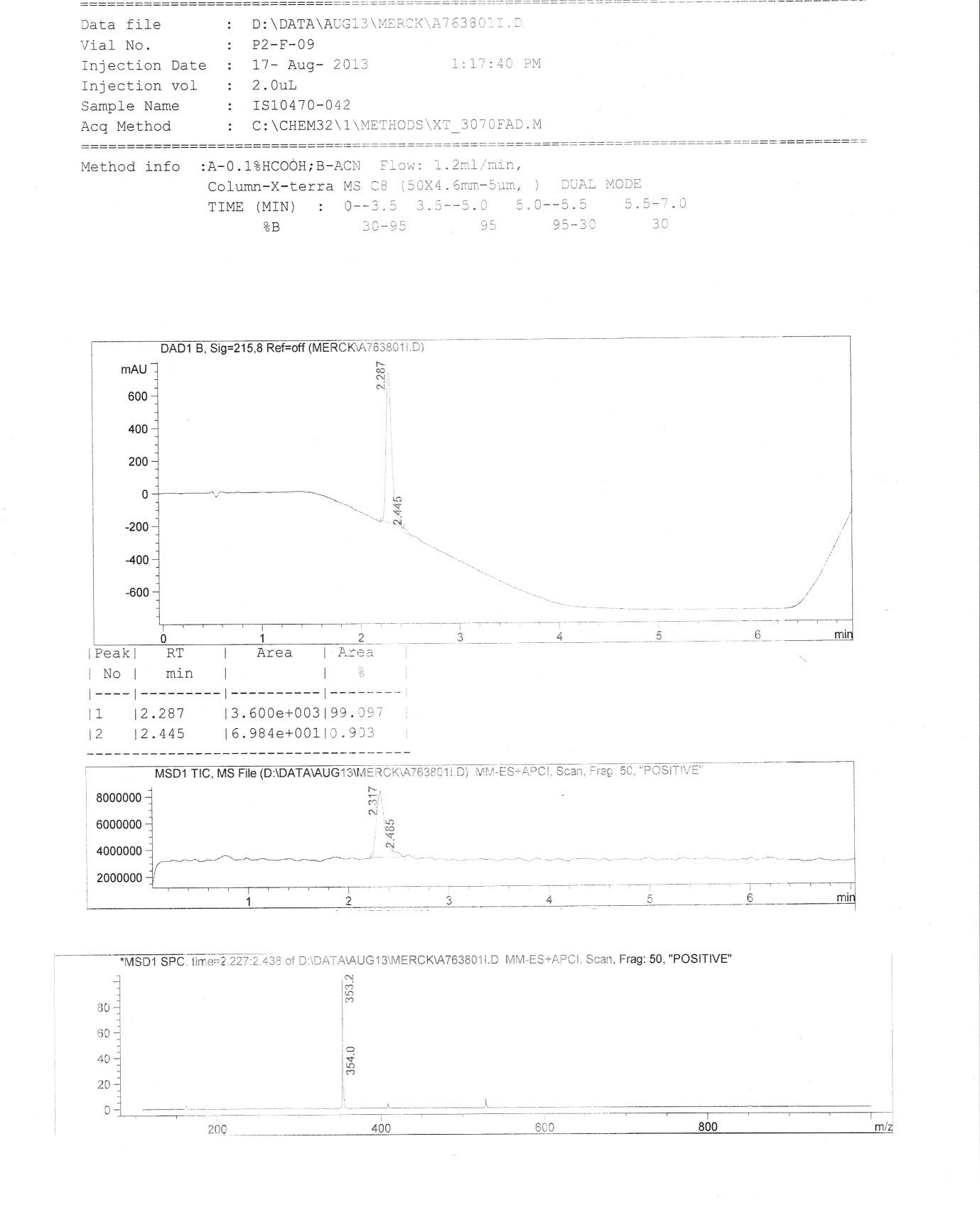

(4g)

***3-(3-phenyl--[1,2,4]triazolo[3,4-b][1,3,4]thiadiazol-6-yl-)phenol (4g)***


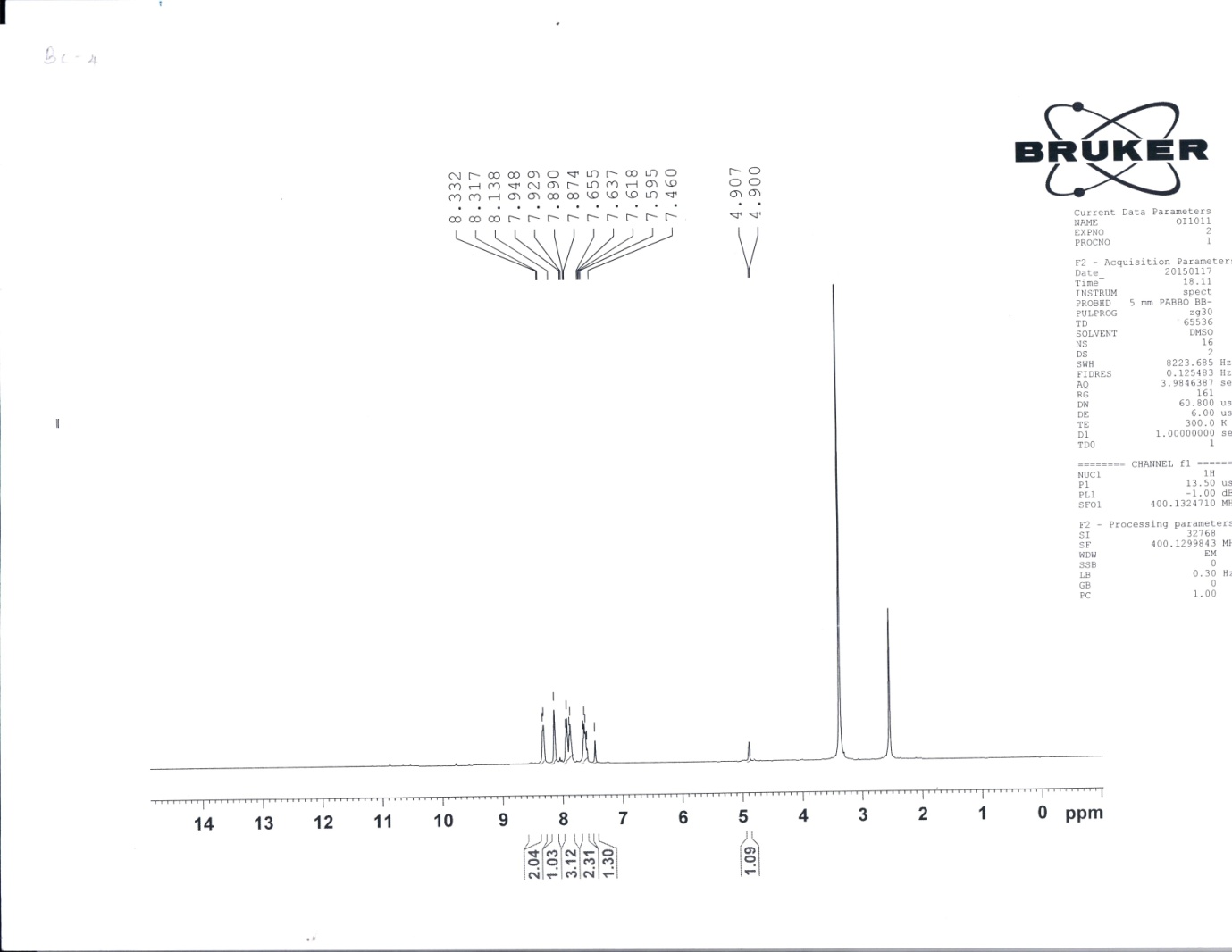


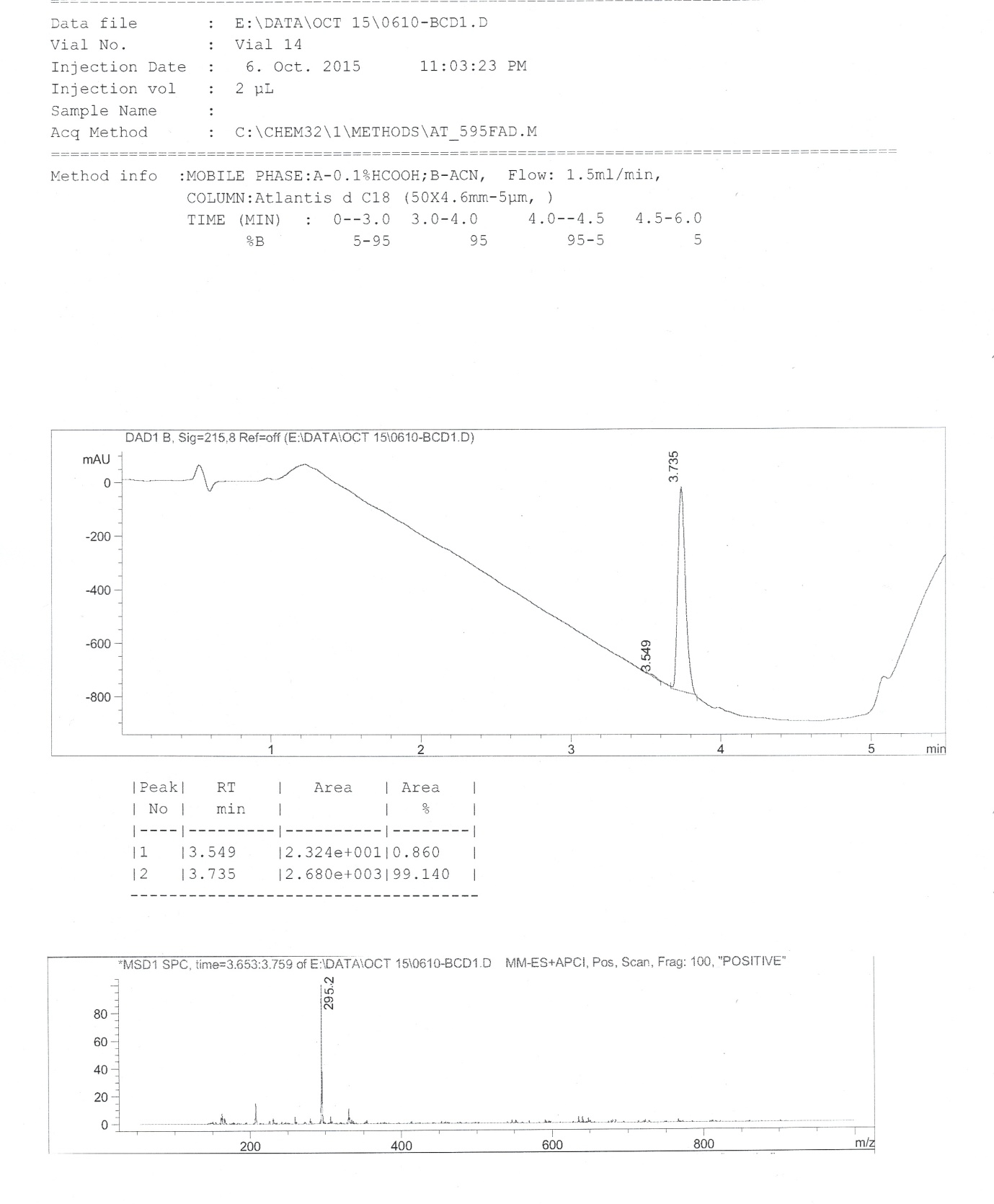

(4h)

***3-phenyl-6-styryl-[1,2,4]triazolo[3,4-b][1,3,4]thiadiazole (4h)***


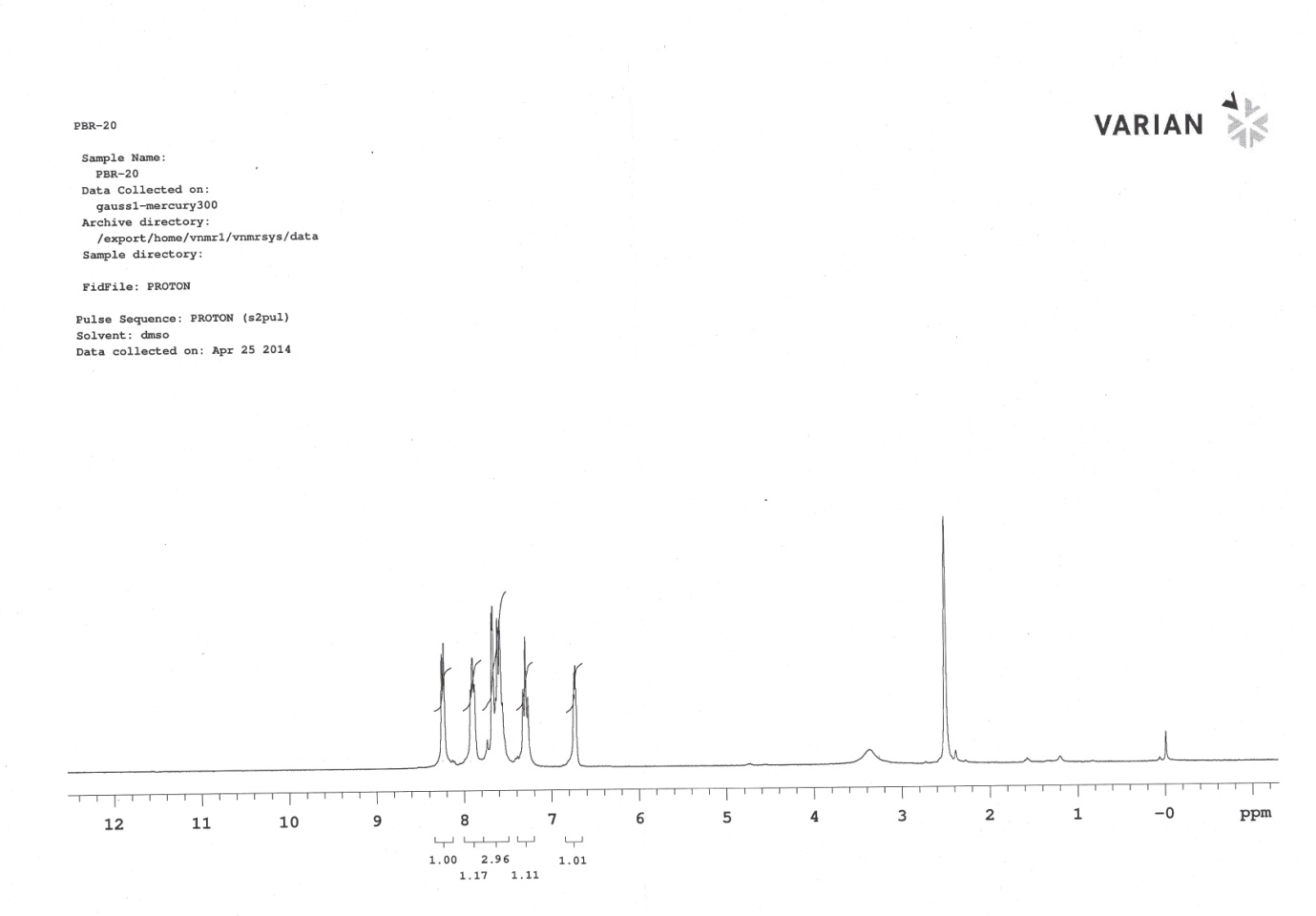


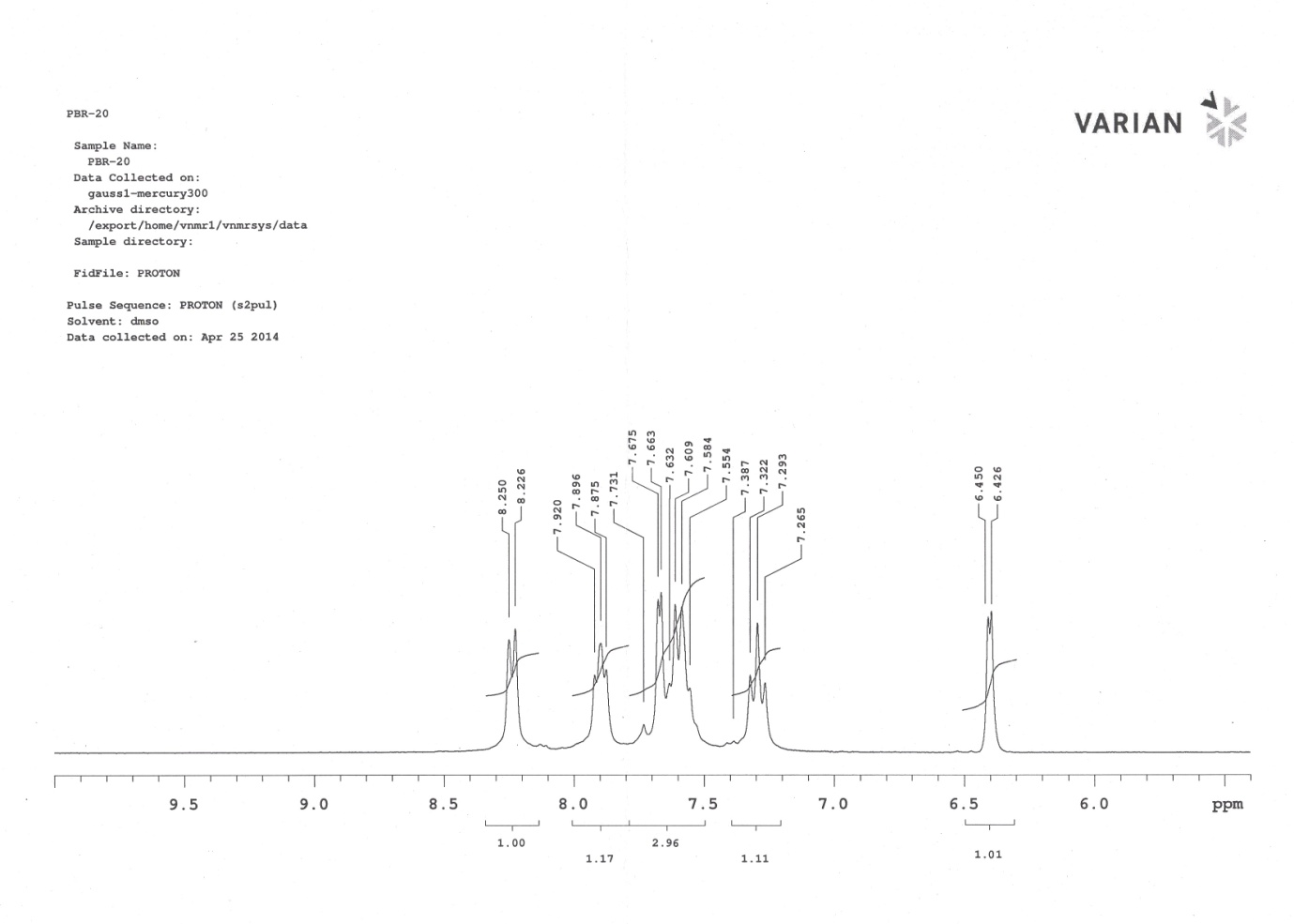


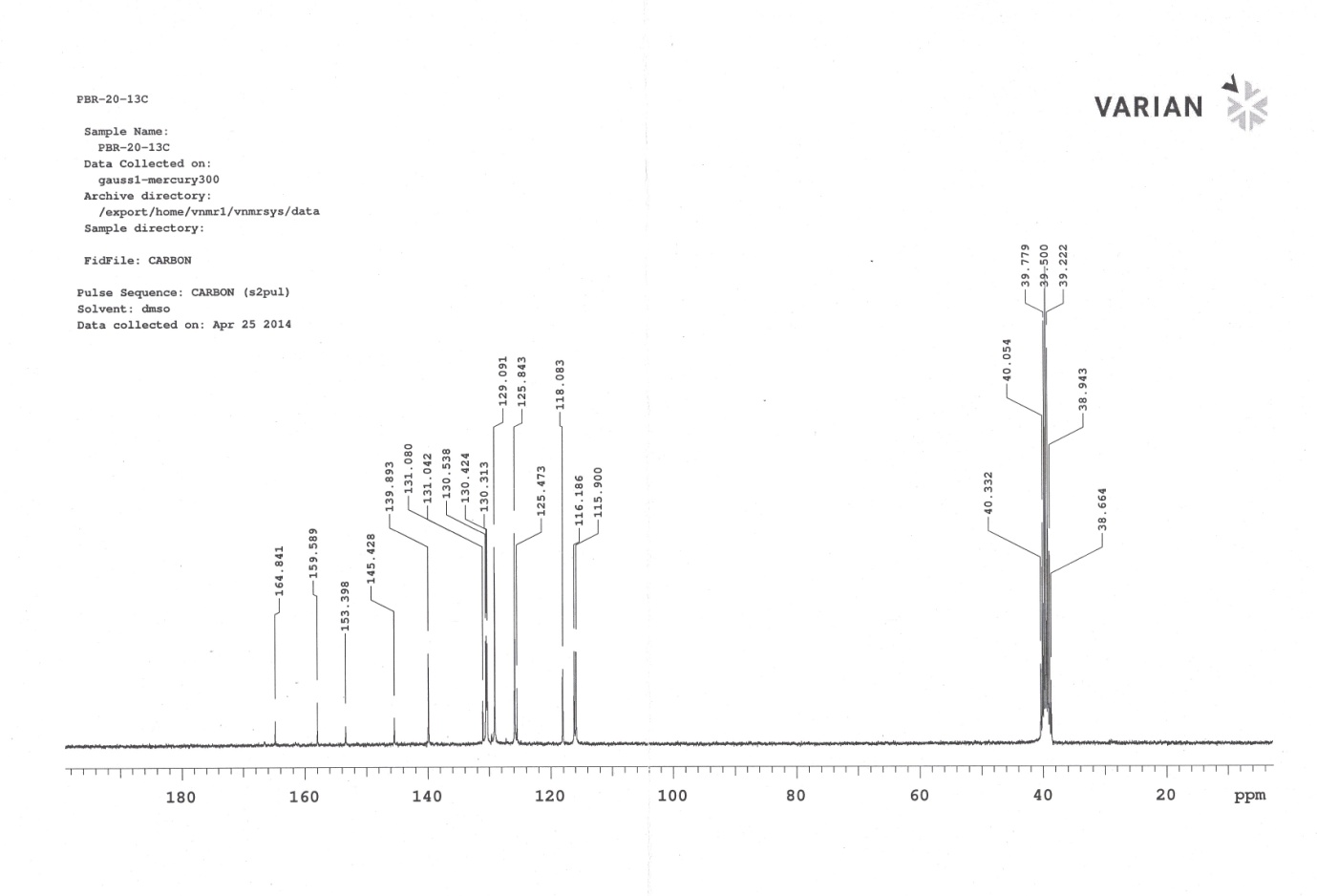


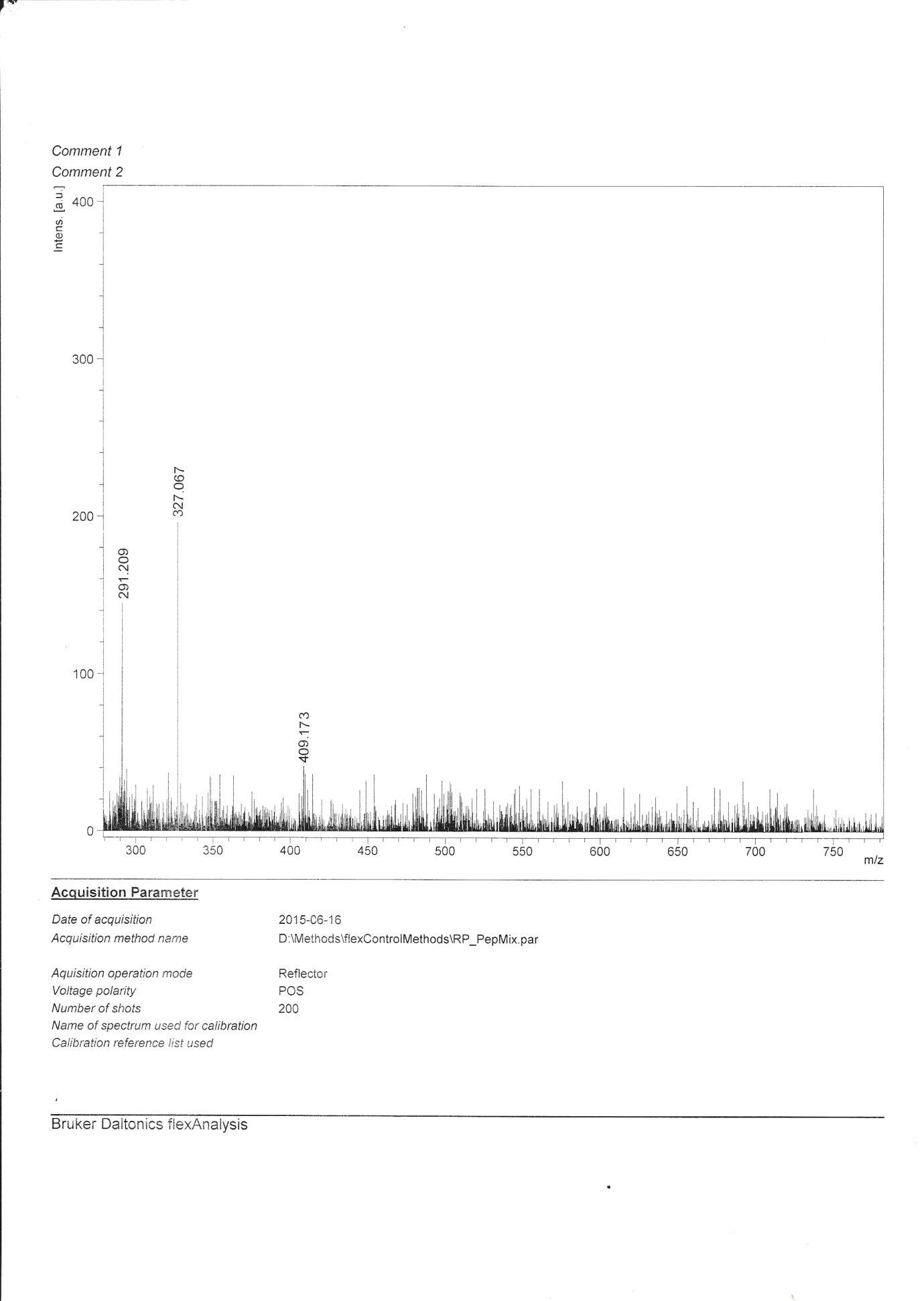

(5a)

***2-hydroxy-3,5-diiodo-N-(3-phenyl-5-thioxo-1H-1,2,4-triazol-4(5H)-yl)benzamide (5a)***


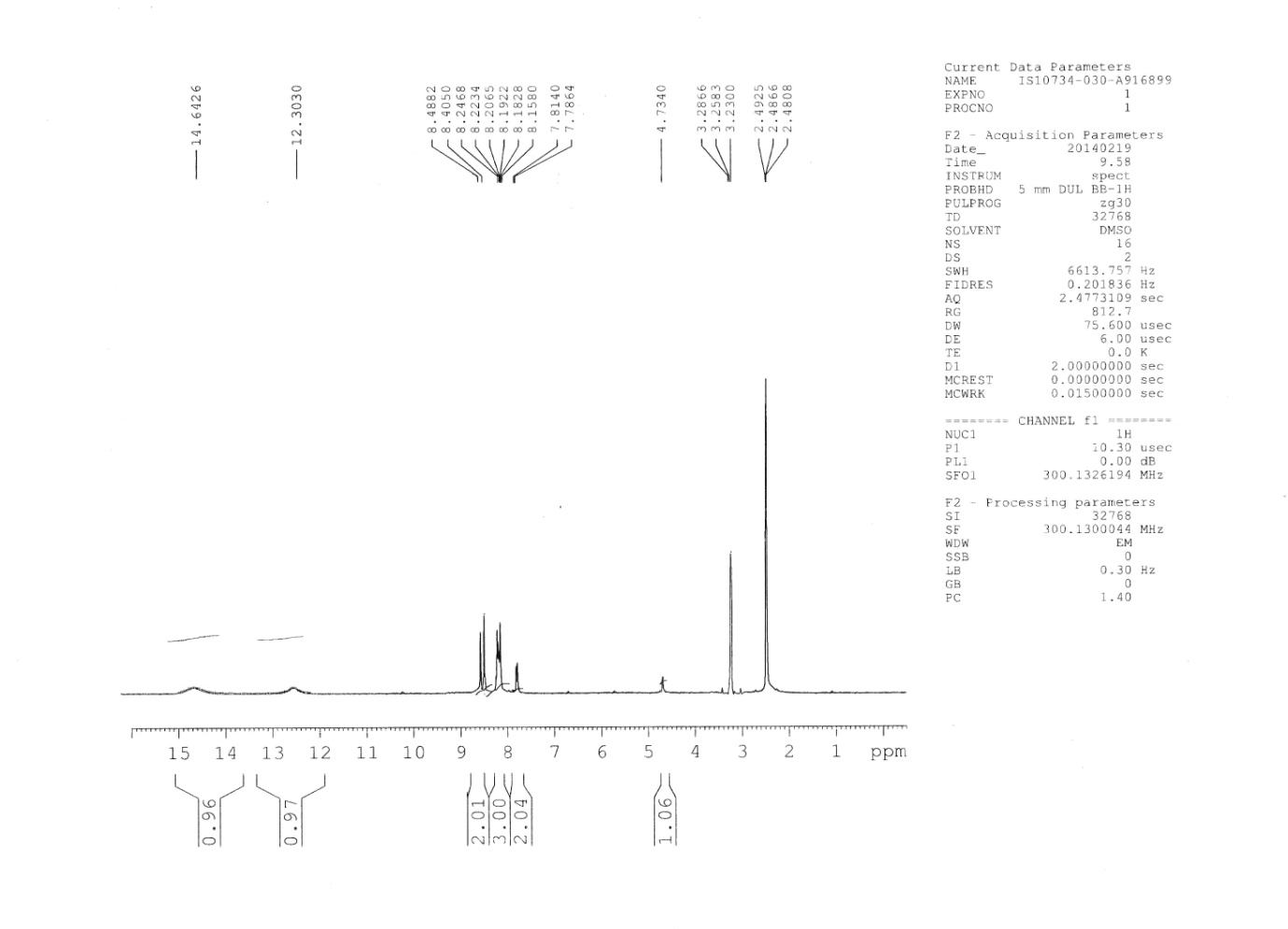


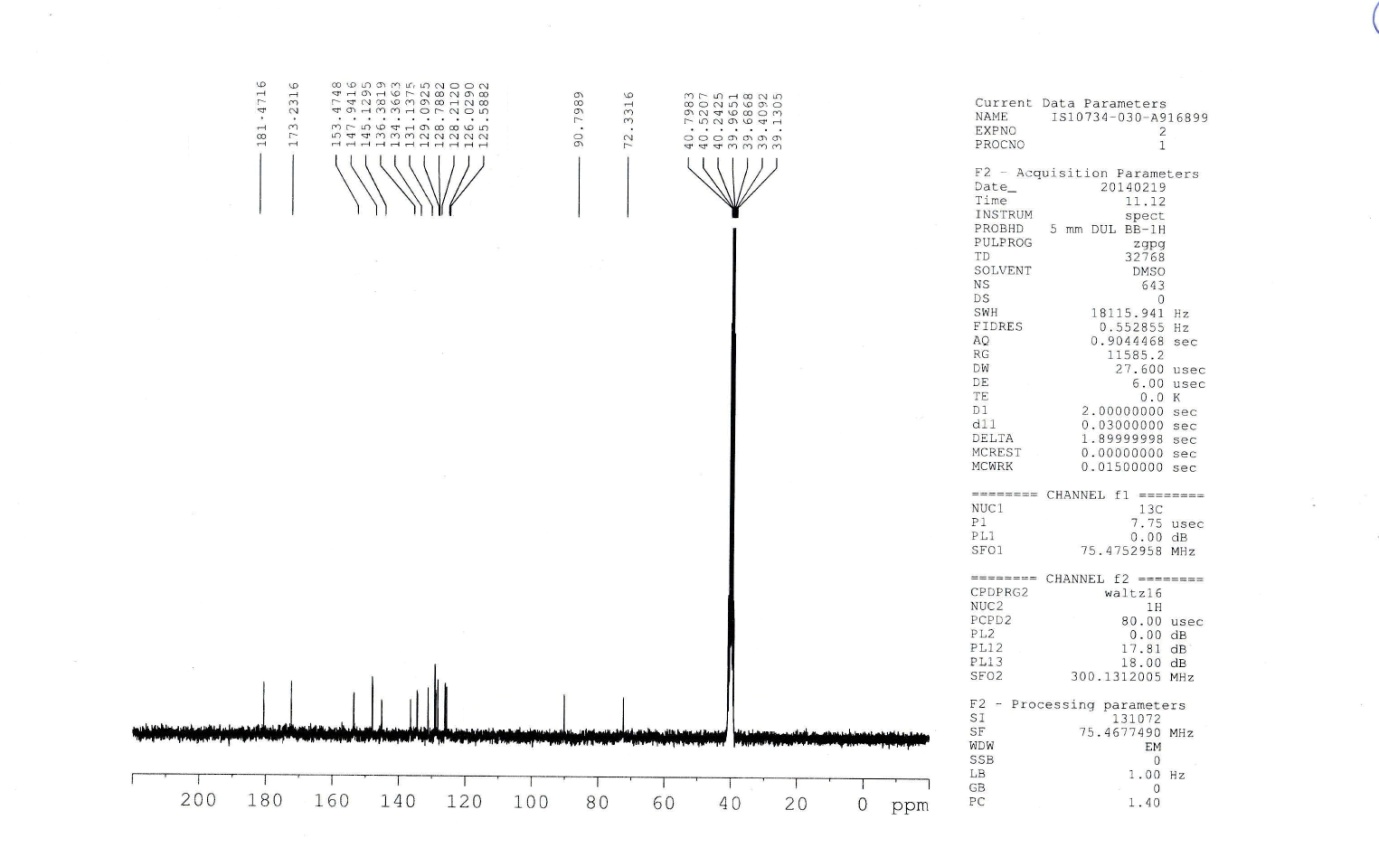


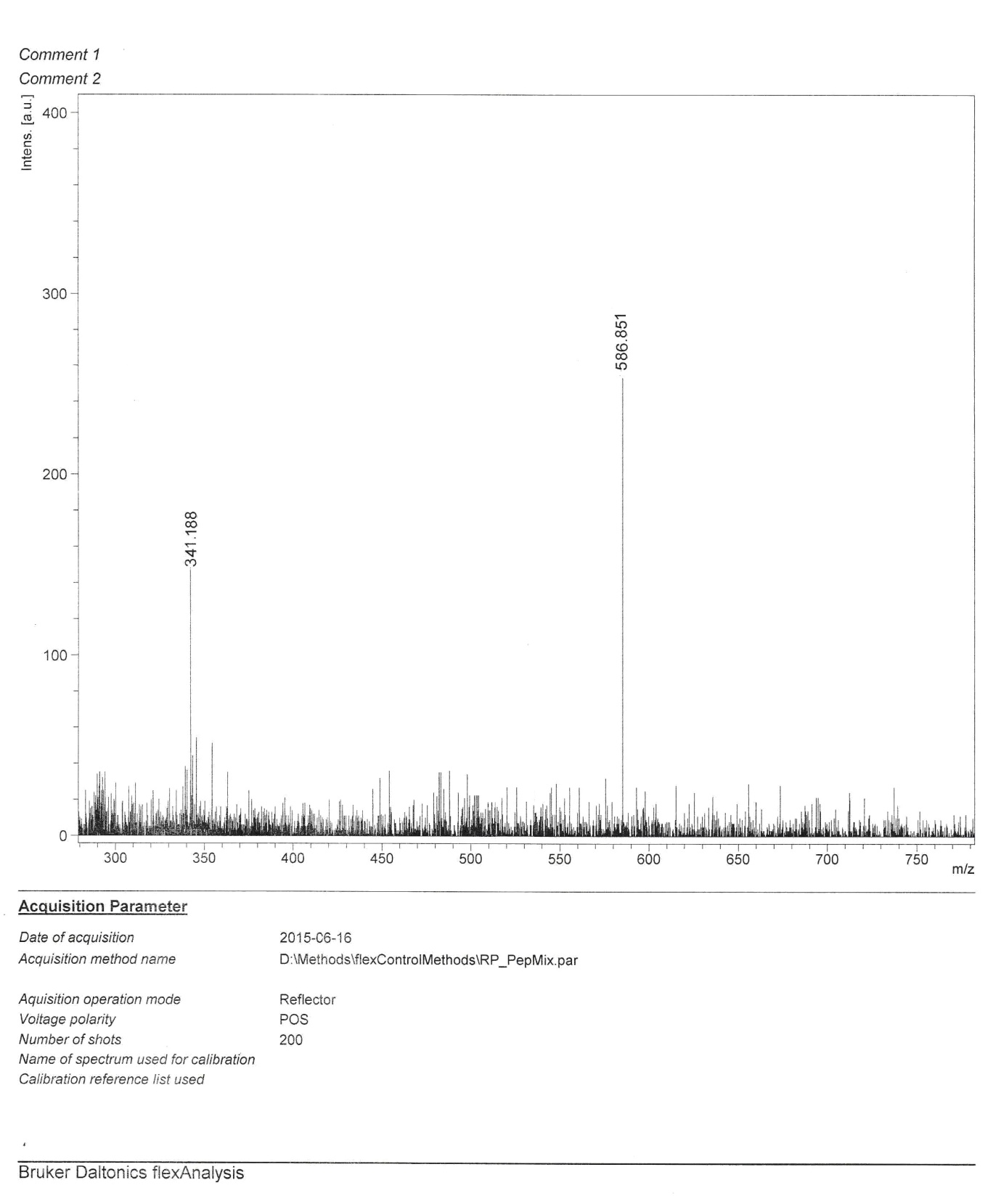

(5b)

***2-hydroxy-5-iodo-N-(3-phenyl-5-thioxo-1H-1,2,4-triazol-4(5H)-yl)benzamide (5b)***


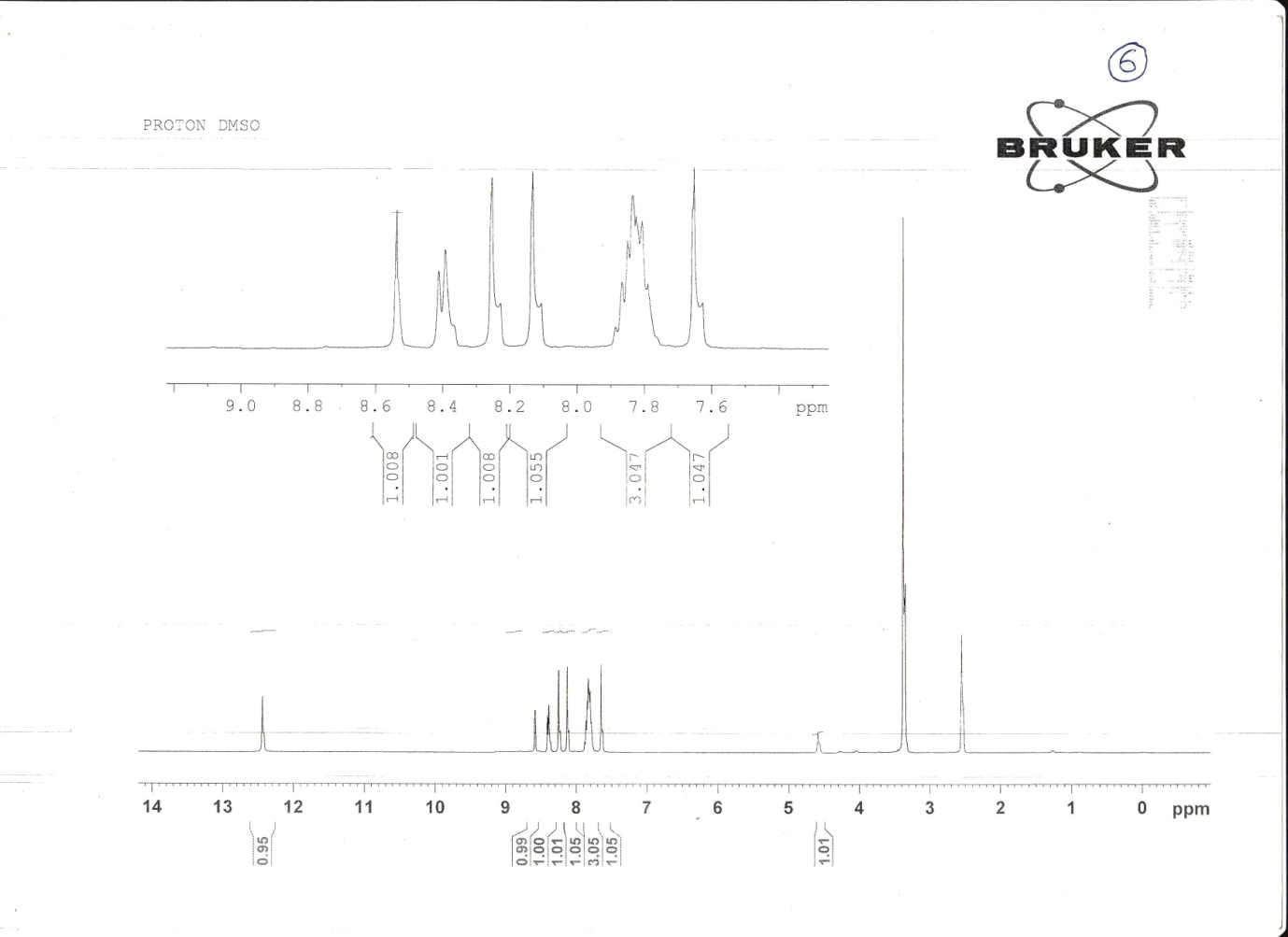


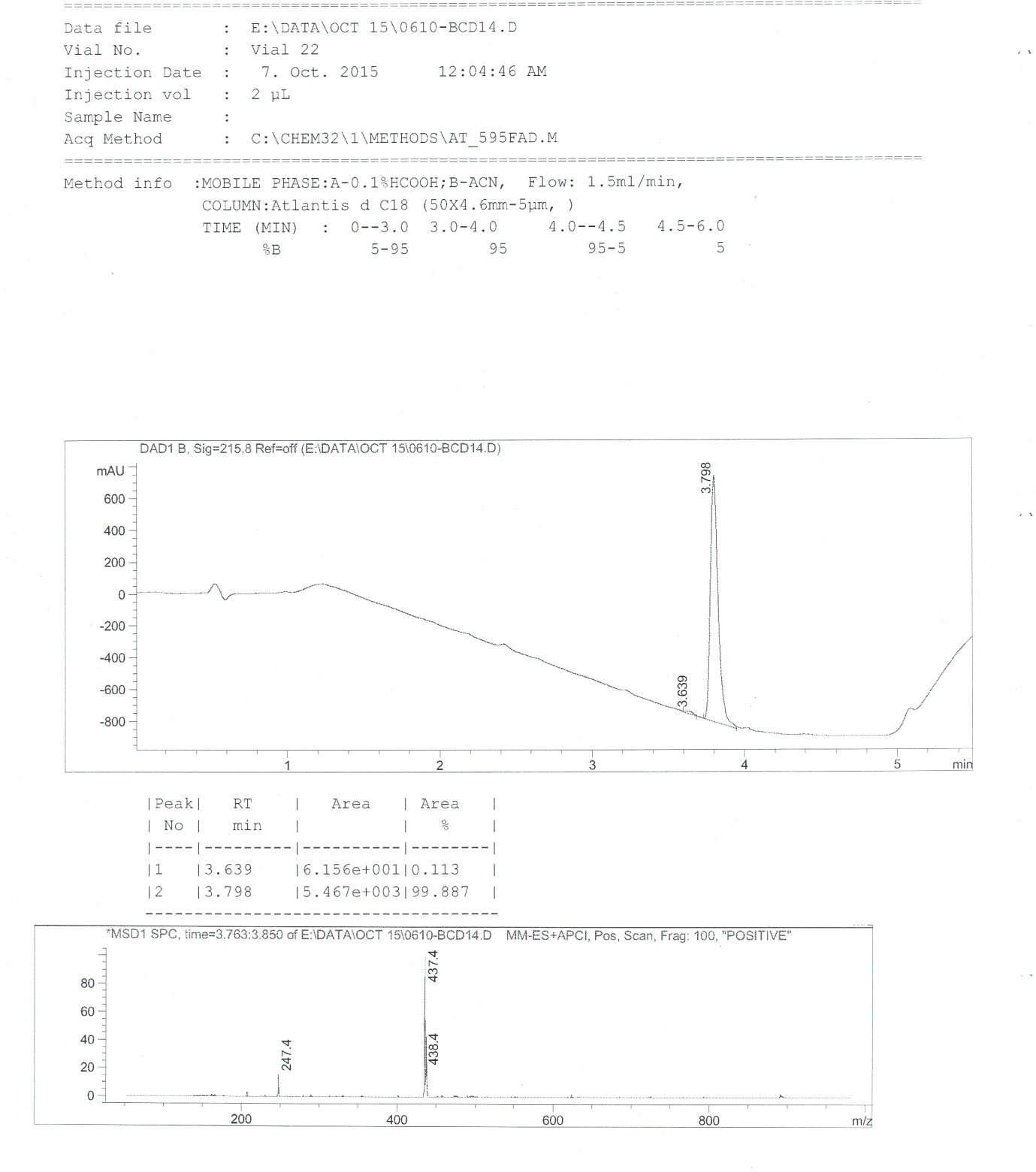

Supplement: Additional file 1: Table S1. — Optimisation of mol% of SCe catalyst, and selection of medium for cyclization reaction. To optimize the reaction conditions for the synthesis of novel 1,2,4-triazolo-1,3,4-thiadiazoles, the reaction was performed in combination of 4-amino-5-phenyl-4 h-1,2,4-triazole-3-thiol and 3-oxo-3-(p-tolyl)propanoic acid as a model reaction in different concentrations of SCe and solvent. The optimal system for cyclization was 20 mol% of SCe in DMF. Table S2. Evaluation of the reuse of SCe for cyclization reaction. The recyclability of the SCe system was evaluated by employing 4-amino-5-phenyl-4 h-1,2,4-triazole-3-thiol with 3-oxo-3-(p-tolyl)propanoic acid to yield 2-(3-Phenyl-[1, 2, 4]triazolo[3,4-b][1, 3, 4]thiadiazol-6yl)-1-p-tolylethanone. The catalyst was removed by filtration after each run and thoroughly washed with acetone, dried and activated at 823 K and taken for the next cycle. There was a significant reduction in the yield of the product after the second run using SCe. Figure S1. Spectral data. Scanned copy of 1H NMR, 13C NMR, and mass spectra of the indicated compounds. (DOCX 4202 kb) [file 12885_2017_3214_MOESM1_ESM.docx]
